# Drug Delivery Strategies for Avobenzone: A Case Study of Photostabilization

**DOI:** 10.3390/pharmaceutics15031008

**Published:** 2023-03-21

**Authors:** Amol D. Gholap, Sadikali F. Sayyad, Navnath T. Hatvate, Vilas V. Dhumal, Sagar R. Pardeshi, Vivek P. Chavda, Lalitkumar K. Vora

**Affiliations:** 1 Department of Pharmaceutics, St. John Institute of Pharmacy and Research, Palghar 401404, Maharashtra, India; 2 Department of Pharmaceutics, Amrutvahini College of Pharmacy, Sangamner 422608, Maharashtra, India; 3 Institute of Chemical Technology Mumbai, Marathwada Campus, Jalna 431213, Maharashtra, India; 4 Department of Pharmaceutics and Pharmaceutical Technology, LM College of Pharmacy, Ahmedabad 380009, Gujarat, India; 5 School of Pharmacy, Queen’s University Belfast, 97 Lisburn Road, Belfast BT9 7BL, UK

**Keywords:** encapsulation, avobenzone, drug delivery, sunscreen, photostabilization

## Abstract

Several developments and research methods are ongoing in drug technology and chemistry research to elicit effectiveness regarding the therapeutic activity of drugs along with photoprotection for their molecular integrity. The detrimental effect of UV light induces damaged cells and DNA, which leads to skin cancer and other phototoxic effects. The application of sunscreen shields to the skin is important, along with recommended UV filters. Avobenzone is widely used as a UVA filter for skin photoprotection in sunscreen formulations. However, keto-enol tautomerism propagates photodegradation into it, which further channelizes the phototoxic and photoirradiation effects, further limiting its use. Several approaches have been used to counter these issues, including encapsulation, antioxidants, photostabilizers, and quenchers. To seek the gold standard approach for photoprotection in photosensitive drugs, combinations of strategies have been implemented to identify effective and safe sunscreen agents. The stringent regulatory guidelines for sunscreen formulations, along with the availability of limited FDA-approved UV filters, have led many researchers to develop perfect photostabilization strategies for available photostable UV filters, such as avobenzone. From this perspective, the objective of the current review is to summarize the recent literature on drug delivery strategies implemented for the photostabilization of avobenzone that could be useful to frame industrially oriented potential strategies on a large scale to circumvent all possible photounstable issues of avobenzone.

## 1. Introduction

**Figure 1:**
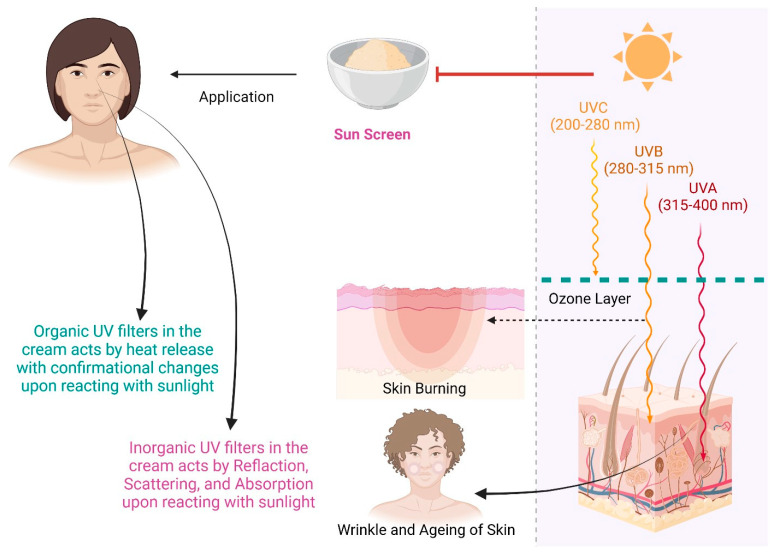
Schematics of the utilization of sunscreens to protect against damaging impacts of sunrays wherein UV rays are responsible for noxious effects attributed to sunlight. Three bands make up the UV region, which has a wavelength range of 100–400 nm: UVA (315–400 nm), UVB (280–315 nm), and UVC (100–280 nm). The ozone layer is currently shielded from UVC rays. High-intensity UVB radiation causes more rapid harm than low-energy UVA radiation, which can penetrate further into the skin and cause long-term impact. Utilizing inorganic UV filters can create a barrier that keeps UV rays from penetrating the skin. This barrier could either reflect or scatter light.

Ultraviolet (UV) filter substances prevent ultraviolet light from passing through the skin [1]. There are two types of UV filters used for photoprotection. Dermatologists classified UV filters as inorganic and organic agents [2]. Organic filters absorb a narrow band of ultraviolet radiation (UVR), whereas for inorganic filters, the interaction of absorption and scattering results in “wide spectrum” (UVA and UVB) protection (Figure 1). These sunscreen filters are used to guard the skin against harmful skin radiation and erythema by reflecting or absorbing UV rays. These agents are also employed to protect against photodegradation in cosmetics, such as makeup, and other industries, such as plastics and paints [3]. To measure the amount of photoprotection provided by these agents, the sun protection factor (SPF) is used. This SPF shows sunscreen efficacy that is mostly related to how much protection is facilitated by a given UV filter [4].

On the other hand, most commercially available sunscreen chemicals exhibit photoreactions that result in the formation of harmful products [5]. It was found that the sunscreens AVOB and Ecamsule (ECAM) were unstable. Sunscreens safeguard the skin from the damaging impacts of UVR, such as DNA damage, photoaging, and sunburn. Regular UV exposure to human body parts can cause considerable skin damage. UVC, UVB, UVA1, and UVA2 are the four subgroups of UV light [6], as shown in Figure 1. The atmosphere absorbs UVC radiation, which does not affect the Earth’s surface. However, UVB and UVA rays affect the earth’s surface and damage human skin. As a result, UVB and UVA rays are either absorbed or reflected by sunscreen filters. UVB has been demonstrated to damage the skin’s upper surface but does not penetrate the skin; therefore, there is no sunburn or DNA damage. UVA light penetrates deeper into the skin, causing photosensitized oxidation of DNA molecules. Two types of processes are involved in this process: type ‘A’ and type ‘B’ [7]. Once an active sensitizer combines with oxygen, type “B” promotes the creation of singlet molecular oxygen; in contrast, type “A” produces a radical in an active state through electron or hydrogen atom transfer. There are two types of UV-light filters: chemical and physical; the authors have mainly discussed chemical filters. Chemical filters come in various varieties, including UVA and UVB filters as well as broad-spectrum filters that can filter both kinds. Based on their operating methods, sunscreens can be divided into three categories:

- UV filters, such as oxybenzone, are based on excited-state intramolecular proton transfer (ESIPT);
- Filters that necessitate the use of photo stabilizers such as AVOB;
- UV filters are based on reversible photoisomerization, such as emulsions.

There are three components in commercially available sunscreens. Abdul Rahman Abida et al. studied the photostability and photosensitizing capabilities of (a) oxybenzone, (b) AVOB, and (c) Ecamsule [1].

Sunscreens are essential for preventing skin cancer and protecting against photoaging. Ultraviolet filters are generally photosensitive, particularly regarding their carriers, and because they are lipophilic, they might react with plastic packaging. Finally, routine usage of the product at high temperatures can have a substantial impact on UV filter stability. This research aims to determine how stable sunscreen formulas are in polyethylene packaging. UV filters were chosen in both free-form and encapsulated forms. Stability tests were carried out on the packaging and the compositions [8]. Sunscreens are essential for preventing skin cancer and protecting against photoaging. Ultraviolet filters are generally photosensitive, particularly regarding their carriers, and because they are lipophilic, they might react with plastic packaging. Finally, routine usage of the product at high temperatures can have a substantial impact on UV filter stability. The goal of this research is to determine how stable sunscreen formulas are in polyethylene packaging. UV filters were chosen in both free-form and encapsulated forms. Stability tests were carried out on the packaging and the compositions. The findings demonstrated that all the pack’s elastic/plastic behavior and exterior color drastically altered following solar irradiation. Moreover, the researchers learned that the formulation’s UV filter, which is contained in a high-density/low-density polyethylene blend, can deteriorate with time, reducing the product’s effectiveness as a skin protector [9].

AVOB is permitted as a UVA filter in the United States and Europe. This compound, however, cannot be employed with some sunscreens due to its photochemical instability. Researchers looked at the examined absorption and emission spectra, as well as their photochemical degradation, free radical formation, and singlet oxygen photoproduction, among other things. As a result of the investigation, two compounds with UVA-filtering properties were discovered (2-hydroxy-4-methoxychalcone and 2-hydroxy-4-methoxydibenzoylmethane). More investigation is needed, including blending the substances with UVB filters and placing them in emulsions or other frequently used cosmetic forms.

Additionally, the author calculates its toxicological profile. Moderate exposure to sunlight provides several health benefits for humans. Excessive exposure to UV light, on the other hand, can result in burns, photoaging, skin cancer, and other issues. These ingredients are now found in a wide range of skincare products. According to regional regulations, many chemicals are certified as sunscreens for humans. Shaath and colleagues claimed that although fifty-five UV filters were authorized in various regions of the world in 2010, only ten of them were internationally accepted. Presently, there are two categories of UV filters: organic and inorganic. ZnO and TiO_2_ are inorganic filters. The structure of organic filters is used to classify them. There are various vital considerations to consider, such as photostability, toxicity, and final disposal into the environment [10]. However, due to its photochemical instability, this molecule cannot be used in conjunction with some sunscreens. According to recent research, irradiating AVOB causes the molecule to break down into radicals, which produce chemicals such as arylglyoxals and benzyls and react with other sunscreens. It takes an atom of hydrogen from another molecule (generally the solvent). Arylglyoxals are effective photosensitizers, according to research on the biological properties of photodecomposition products.

Ultraviolet rays are considered carcinogens. These rays act as tumor initiators, promoters, mutagens, and nonspecific destructive agents [11]. UV light is abundant in the environment and is also one of the most important elements in skin cancer. These UV rays are also involved in some environmentally influenced skin issues [12]. On the other hand, UV rays help improve human health by facilitating vitamin D and endorphin synthesis in the skin. Nevertheless, excessive exposure to UV rays causes atrophy, wrinkling, pigmentary changes, and even cancer [13]. Each year, many people worldwide are diagnosed with various types of skin cancers. These cancers are linked to UV exposure through epidemiological and molecular level factors. Skin fairness, UV damage, and an increase in skin cancer cases are related to polymorphisms in the specific gene melanocortin 1 receptor gene (MC1R) [14,15]. A better UV protective strategy will be developed through a greater understanding of MC1R function in genome maintenance and molecular mechanisms after UV exposure [16].

Sunscreen filters have a pivotal role in the cosmetic industry. Many marketed sunscreen products are used globally to care for skin-related issues. Many of them contain FDA-approved UV filters combined with AVOB and may have crucial business potential in the near future. Products such as Chaptex Lipcare, Ekran, Glonik Lot, Louv, Maeve, Maskosun, and Melaglow combine UV filters to increase patient care. The available UV filters are imperfect in combatting sun rays. There is a need to have more inventions in the sunscreen industry to prohibit the harmful effects of UV rays [17]. The cases of skin cancer are increasing daily worldwide, so there is a need to address these issues with a proper understanding of present scenarios in the sector, available resources, chemistry, drug delivery systems, and photo strategies for photounstable drugs. These will help to gain an overall synergistic impact of photostabilization in the cosmetic industry.

Several UV filters, such as avobenzone (AVOB), absorb primarily in the UVA region. AVOB has an extremely high molar absorption coefficient. It is also called butyl methoxydibenzoylmethane, and it undergoes keto-enol tautomerism [18]. AVOB has a maximum absorption of 357 nm. It is a widely used UVA filter; however, it is unsuitable with other ultraviolet filters due to photoinstability. The UV filter interacts with the energy of electromagnetic waves through absorption or scattering. The dispersion of inorganic particles played a role in this process. These inorganic particles can absorb UV radiation. In the European market, these products are generally used for the same. Many more widely accepted UV filters are required in the creation of improved sunscreen to combat the global rise in skin illness [19]. The UV absorber photostability is measured with reference to the ability to withstand light. After absorption of UV light, the UV filter molecules undergo certain photochemical processes that degrade both their physical and chemical properties. This results in undesired reactions and byproduct formation. Currently, AVOB is widely used as a UVA filter, which degrades reactive species after the absorption of excessive UV radiation [20].

Currently, there are a finite number of UV filters approved by regulatory agencies, and AVOB is one of them currently used in sunscreen formulations. To the best of our knowledge, no review has focused on strategies implemented to address AVOB photostability issues. We discuss the photostability of AVOB in sunscreen products in this article. We attempted to provide an update on the current status, research trends, and strategies for novel AVOB drug delivery systems.

## 2. Photoprotection and UV Filter Importance

For human safety, the photoprotective product’s photostability or photoinstability is commonly described in negative terms. Some sunscreen products infrequently cause photo irritation and photoallergic reactions compared to undesirable side effects, such as skin irritation. There is more invention required to explain the stability of UV filters concerning photons and study the acute and chronic toxicological effects of UV filters on humans [21]. Many organic ultraviolet filters tend to photodegrade; therefore, specific designs are applied to achieve high efficacy and strategies to maintain and promote photostability. Therefore, concrete improvement in the photostability of sunscreen products remains a significant goal for producers [17].

## 3. Ultraviolet Filter Photostability Issues

UV filters are found in many sunscreen products. When these products are exposed to sunlight, the chromophore will absorb the energy given by sunlight. It undergoes an excited state. The amount of energy absorbed by the molecule is dependent on the extent of photo exposure. The absorbed energy is dissipated by a UV filter [22]. In many cases, this absorbed energy is released relatively quickly in a few seconds. When the UV filter releases absorbed energy, it returns to its original state from the excited state through fluorescence or phosphorescence. There are several different ways to dissipate the absorbed energy, such as fragmentation, isomerization, reaction with other molecules, and generation of free radicals [23]. UV filter deterioration is the key reason for such reactions. Sunscreen products require photostability and should be provided continuously rather than with an all-or-nothing response.

Several factors are considered for the photodegradation of UV filters, such as fluency, heat, time, and application density [24]. To evaluate the efficiency of photostability, different methodologies, such as absorbance/transmittance, are used. The quantitative evaluation of each UV filter is fairly difficult in analytical methods and provides information on the few photoprotective values. Therefore, different manufacturers have to rely on UV absorption tests to measure the stability of UV filters both alone and in conjugation with different filters [17].

## 4. Importance of Photostabilization for Photosensitive Drugs

UV light plays a very important role in health products, as they are exposed to the same light through production, storage, distribution, and patient use [25]. Some chemicals are light-sensitive and are usually affected by light. When exposed to light, such chemicals lose or reduce their therapeutic action and may form hazardous products as a health risk. Such chemicals are photostable by advanced topical systems [26]. Many photosensitive chemicals, such as tretinoin, are preferred for the management of skin illnesses such as psoriasis, acne vulgaris, and certain neoplasias. This chemical can cause substantial skin damage, such as phototoxic/photoallergic reactions, due to its photo instability. For the oral and parenteral administration routes, photostability is achieved by utilizing proper packaging materials, avoiding sunlight exposure after administration, or using sunscreens [27]. Several photostabilization strategies, such as the use of antioxidants, encapsulation of photosensitive drugs in lipidic nanoparticles, microparticle formation, and inclusion complexation of drugs, have been developed [28].

Photoprotective techniques can include light-resistant packaging or suitable coatings. The photostability of topical medicines must be maintained to safeguard active components during high exposure to sun rays. Several strategies have been implemented for the photostabilization of AVOB and are included in the literature review. Encapsulation is the most recommended approach, followed by the inclusion of antioxidants and photostabilizers. Cyclodextrin was one of the most commonly used photoprotective barriers, followed by the formation of liposomes, lipid nanoparticles, and microparticles. In a few cases, other approaches have been applied, such as the preparation of niosomes and mesoporous silica nanoparticles for effective photostabilization of photosensitive drugs [29].

## 5. Novel Strategies Used for Photostabilization and Photoprotection

The photoprotection delivered by sunscreen agents is essential for customers to take care of highly intense sunrays and decrease the burden of phototoxicity issues arising from the same. The packaging material itself is insufficient to protect the active drug, as in the case of the topical formulation; similarly, sunscreen agents must be assisted by UV filters to make them effective for a broad UV spectrum. AVOB has been photostabilized using a variety of methods depending on the phototoxicity issues, photodegradation mechanism, and compatibility of UV filters with added excipients. There are four major strategies used to achieve photostabilization for photounstable UV filters: encapsulation, implementation of antioxidants, utilization of photostabilizers, and quenchers. The encapsulation strategy involves using several polymers, such as cyclodextrin with antioxidants, including morin, to obtain possible synergistic effects through novel drug delivery systems, such as lipospheres. Some researchers have used octocrylene as a photostabilizer for better photostabilization of avobenzone. More exploration is required to develop suitable quenchers to induce proper photostabilization of avobenzone [20]. Only a few of the compounds have shown effective photostabilization as ROS (reactive oxygen species) trappers, UV pearls (encapsulated sunscreens), microparticles (hollow styrene-acrylate copolymer spheres), and blockers of the triplet-triplet and singlet-singlet mechanisms. UV protection is lost as a result of photodegradation because the resultant photoproducts absorb UV light at shorter wavelengths than their parent compounds [30]. The toxicity of sunscreens as a result of their breakdown products is an important factor to address at the skin or percutaneous absorption level. The approach is not to use photochemically reactive substances that need extra molecules to protect them in a cosmetic composition [31]. Robert M. Sayre et al. used films or other UV filters to protect AVOB [32]. Murphy and colleagues looked at how deuteration affected the balance of keto-enols and the stabilities of the UV filter AVOB [33].

## 6. Avobenzone Drug Profile

The details of the avobenzone properties are given in Table 1.

### 6.1. Structure and Tautomerism of Avobenzone

**Figure 2:**
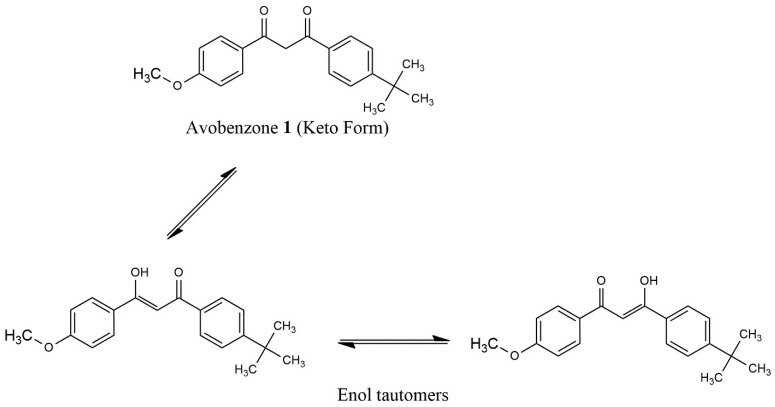
Structure and keto-enol tautomerism of avobenzone.

In the AVOB solution, the enol form and keto form are present in equilibrium. In solution, two isomeric forms of the enolic form exist. The keto form has a butterfly-like structure, and the enol form is planar. Delocalization of the enolic form is due to the methoxy group. The H-bonding is resonance-helped in the chelated enol form, and the synergistic impact of pi delocalization and H-bond creation results in robust H-bond formation. In some solvents, photolysis converts the enol to the keto form, which eventually returns to the enol form in the dark, as shown in Figure 2.

Two bands are obtained. The enol form of AVOB produces a band at 355 nm, and the keto form of AVOB in cyclohexane produces a band at 265 nm. In more polar solvents, the enol band shifts to higher wavelengths: ethyl acetate = 356 nm, DMSO = 363 nm, and methanol = 358 nm.

The phosphorescent stage of the enol lasted for 30 milliseconds. AVOB fluoresced if diluted in ethyl acetate at ambient temperature, with fluorescence peaks at 405 nm, a maximum yield of 0.01, and a fluorescence lifespan of 13 ps. While the triplet state only lasts for approximately 190 milliseconds, the keto form exhibits strong phosphorescence in the range of 410–450 nm. The keto form is only marginally luminous. The triplet state has no EPR signal. The AVOB photoinstability is related to the long-lived triplet state of the keto form.

### 6.2. Steady-State Photolysis

AVOB photolysis is affected by both the solvent and the wavelength. The literature in this field is quite variable, owing to differences in irradiation circumstances, flux, wavelength, and concentrations used [34]. According to consensus, AVOB absorption spectra are more variable in nonpolar solvents than in polar solvents. The phototautomerization of the enol to the keto form causes a change in AVOB [35]. In the dark, the keto state reverts to enol. Additional photolysis is conceivable, and it seems that the keto form occurs most frequently [36].

### 6.3. Photodecomposition of Avobenzone

AVOB causes an electron to be excited from the lowest energy to the singlet-exited state by absorbing UV light (photons). Typically, this departed singlet-state electron can either cross to the excited triplet state or return to the ground state, which can cause phosphorescence or heat production or undergo further photodegradation processes. This produces a photoallergic reaction in the skin [37].

AVOB after 100 h of exposure to a mercury lamp (185–400 nm) and the entire photodecomposition of AVOB in cyclohexane was observed. GC-MS revealed three degradation products: p-methoxy benzoic acid, *p*-*tert*-butyl benzoic acid, and t-butylbenzene. Some of the principal photoproducts generated were degraded due to harsh photochemical conditions. The production of benzoic acids could be explained by the fission of one of the two C-C bonds close to the C=O of the keto form. Photodegradation is caused by the photo-excited keto form. C-C bond fission occurs in the triplet state. Photodecomposition is a complicated process that produces a variety of compounds that absorb preferentially at wavelengths less than 260 nm. The ground-state enol and singlet or triplet oxygen produced by the triplet keto form can mix to form oxygenated molecules. Additionally, the triplet keto form can destroy the enol form during photolysis [37,38].

### 6.4. Structure-Activity Relationship (SAR) of Avobenzone

**Figure 3:**
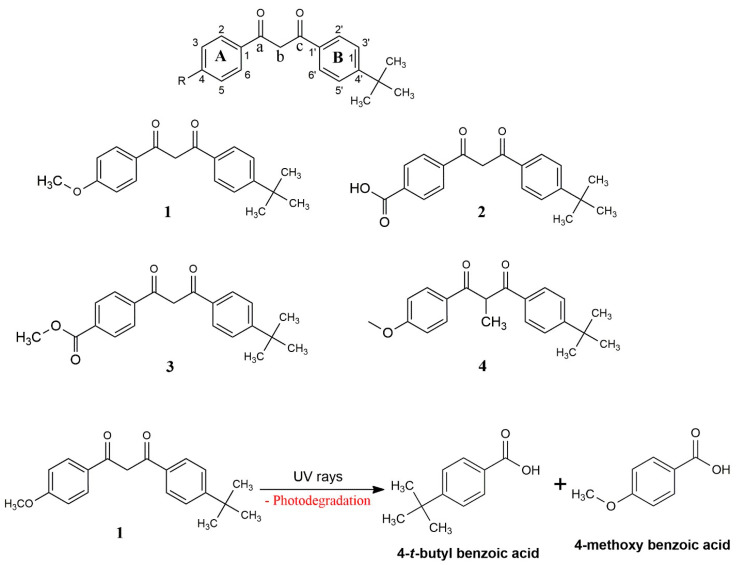
Structure-activity relationship and photodegradation of avobenzone: The substitution of the R group on ring ‘A’ of avobenzone with electron-withdrawing groups increases the photostability, while electron-donating groups destabilize it (entries 1, 2 and 3). Furthermore, blocking the keto-enol tautomerism by replacing the acidic hydrogen with the methyl group also contributes positively to the photostability of avobenzone (entry 4). Avobenzone degrades into 4-t-butyl benzoic acid and 4-methoxy benzoic acid [37,38].

AVOB is chemically diketomethane, which is unstable (responsible for photodegradation) in the keto form but readily converted into the most stable enol in the solution phase. A very nice study on the effect of different functional groups on two different benzene rings (Figure 3) by Raghothama et al. suggested that the electron-withdrawing groups on benzene ring ‘A,’ such as the carboxylic acid (Figure 3, entry 2) and methyl carboxylate (Figure 3, entry 3), increase the photostability of AVOB, while electron-donating groups on the same benzene ring highly destabilize AVOB. In another study by Miranda et al., methylation of the acidic hydrogen at position ‘b’ (Figure 3, entry 4) also increases the photostability of AVOB. The probable reason for the enhancement of the stability is the blocking of the diketo group, which is responsible for the photodegradation of AVOB, as shown in Figure 3. Apart from this, the butylated benzene ring (ring ‘B’) is needed for the activity because it may help to trap the free radicals that are generated by exposure to sunlight. From the above study, it is very clear that the enhancement of the stability toward photodegradation is increased with the electron-withdrawing group, while the electron-releasing group may destabilize AVOB. In the future, it will be interesting to study the effect of other electron-withdrawing groups, such as nitro, cyano, and carbonyl compounds, on the stability of AVOB [36,39].

## 7. Examples of Novel Approaches Used for Enhancing the Properties of UV Filters

Recently, the efficiency of UV filters for people and the environment has been questioned. In reality, numerous investigations have validated the presence of UV filters in biofluids from humans and marine species, suggesting that UV filters have hazardous consequences [21]. In UV filter designs, certain chemical moieties give inherent toxicity. UV filters that photoisomerize or photodegrade experience photoinstability, which can lead to the production of harmful photodegradants and a loss of photoprotective activity [40]. As a result, during the past 20 years, researchers have looked into novel natural materials derived from botanical and marine sources as well as synthetic derivatives in conjunction with nanotechnology, as a method to create new, more powerful, robust, and reliable UV filters [41]. The new generation approaches to enhance the UV filter efficiency include (a) incorporation of antioxidants to combat potentially hazardous photogenerated free radicals [8], (b) use of UV filters (physical, chemical or combination of both), (c) confinement of photoactive compounds in a host via inclusion complexes or carriers (polymeric as well as lipidic), etc. [42].

### 7.1. Singlet Oxygen Quenchers for Photostabilization

Due to preparations containing avobenzone undergoing photofragmentation and producing reactive components (such as free radicals) and reactive intermediates, the photoinstability of avobenzone is not just a photochemistry issue but also a toxicological problem [43]. Avobenzone’s need for stabilization is partially met by presently available photostabilizers, but they are unable to suppress free radicals produced as a result of photofragmentation. In this context, Chaudhari et al. designed a photostabilizer that has antioxidant activity and studied its effect on UV filters. The photostabilizer diethyl hexyl syringylidenemalonate (DESM) was used in different formulations of sunscreens containing different UVA and UVB filters, such as AVOB, homosalate, and octisalate. According to the results, the photostability of AVOB and other UV filters is greatly improved by this photostabilizer, which is also photostable. The photodegradation of AVOB caused by singlet oxygen was reduced by DESM, which was found to be a potent singlet oxygen quencher. Due to its wide range of activities and advantageous formulation properties, DESM is a promising photostabilizer for many personal care products intended for both young and older skin [44].

### 7.2. Photostabilization for Avobenzone

In the search for an effective photostabilizer, Ratan K. Chaudhari et al. developed a novel photostabilizer with sun protection factor-enhancing properties in vivo and demonstrated its application in the development of broad-spectrum sunscreen formulations. The study focused on the creation of a broad-spectrum sunscreen using AVOB as well as a novel photostabilizer based on benzylidene pentanedione chemistry. 3-(3,4,5-Trimethoxybenzylidene)-2-4-pentanedione (TMBP) was synthesized using a condensation process and used in a sunscreen formulation that included AVOB. To compare photostability, researchers have employed current photostabilizers, such as OC. UV spectrophotometric and high-performance liquid chromatography (HPLC) tests were used to assess the photostability of TMBP in a methanolic solution, either alone or in the presence of AVOB. Researchers were able to create the highly powerful photostabilizer trimethoxybenzylidenepentanedione depending on the chemistry of benzylidene pentanedione. The sunscreen properties are raised, and AVOB is stabilized in pharmaceutical preparation, particularly well, by TMBP. It is a prospective addition for use in broad-spectrum photoprotective products because of its efficacy [45].

#### 7.2.1. Bemotrizinol as a Photostabilizer

The UV filter bemotrizinol has excellent avobenzone photostabilizing properties. It has been demonstrated that its capability to absorb UV photons enhances the photostability of sunscreen compositions. In contrast, many products have UV filters and other potent antioxidants in the form of vitamin A derivatives, which help the skin recover from photodamage and remain protected. Additionally, they exhibit some UVR absorption and DNA photoprotective properties. One of the vitamin derivatives, retinyl palmitate, can degrade when exposed to UV light, highlighting the significance of researching novel compounds that can increase vitamin photostability in challenging environments. According to other research, vitamin A, C, and E esters, together with UV filters, decreased skin irritation and vitamin A degradation when exposed to UV light. Carolina Gomes Benevenuto et al. studied UV filters combined with retinyl palmitate (RP). Photostability and phototoxicity experiments were used to determine the phototoxic risk assessment. Bemotrizinol was included in both formulations. The photostability of formulations distributed on a glass plate was tested by subjecting them to ultraviolet irradiation. HPLC analysis was used to evaluate the end products. UV filters and combinations were examined for phototoxicity in vitro using suitable monolayer fibroblast cells. Photoallergy and phototoxicity were investigated using clinical studies (e.g., the photopatch test). In clinical tests, none of the formulations were found to be phototoxic or photoallergic. When paired with RP/benzophenone-3, Bemotrizinol, a UV filter, was discovered to be a better photostabilizer than when paired with RP/AVOB. In vitro phototoxicity experiments revealed that the mixture of RP/AVOB was phototoxic, whereas bemotrizinol reduced the phototoxicity. None of the compositions were discovered to be phototoxic in clinical tests [46].

#### 7.2.2. Diethylamino Hydroxy Benzoyl Hexyl Benzoate (DHHB) as a Photostabilizer

As previously mentioned, the majority of the adverse effects are caused by the excited triplet state of avobenzone’s keto form. The development of photodegradation products such as arylglyoxals and benzils, which are frequently linked to photoallergic and phototoxic reactions, may result from this process [8]. As a result, significant effort is being put into creating novel UVA filters as well as photostabilizers, which are molecules that can increase AVOB photostability by quenching excited AVOB states. In this context, Camila Martins Kawakami et al. used diethylamino hydroxy benzoyl hexyl benzoate (DHHB) as a photostabilizer in a sunscreen formulation containing the UV filter AVOB and evaluated it for photostability. The goal of this study was to examine the photochemical behavior of DHHB and its photostabilizing influence on AVOB in various photoprotective formulations. HPLC and spectrophotometry were used to test the photostability of the compositions. 3T3 fibroblast cultures were used to test in vitro phototoxicity. Ultraviolet A UVA protection was excellent in all of the formulations. HPLC analysis revealed that DHHB had no photostabilizing effect on AVOB. Fluorescence spectroscopy shows that DHHB has no effect on the lifespan of the AVOB excited state and that AVOB and DHHB interact via a static quenching process. Furthermore, energy transfer via the Forster resonance energy transfer (FRET) mechanism, the most prevalent pathway for singlet-singlet quenching, appears to be implausible in this study. These findings help to explain why, in the AVOB singlet excited state, DHHB did not act as a photostabilizer. According to phototoxicity tests, combinations including DHHB have no phototoxic potential. DHHB was revealed to be photostable in all formulations examined but did not affect the photostability of AVOB. As a result, designers conclude that DHHB (F2)-containing formulations are better than AVOB. Under the provided experimental conditions, the results of the HPLC-based photostability analysis demonstrated that DHHB did not increase the photostability of AVOB. In this work, the FRET mechanism, which is the most common cause of singlet-singlet quenching, appears unlikely. Furthermore, DHHB did not impact the individual or average lifetimes of the AVOB singlet excited state. These results provide an explanation for why DHHB did not serve as a photostabilizer in the AVOB singlet excited state [47].

#### 7.2.3. Bis-Ethylhexyloxyphenol Methoxyphenyl Triazine as a Photostabilizer

Chronic UVA exposure causes skin damage, which has been widely established. As a result, current sunscreens should not only shield against UVB and UVA radiation but also sustain that protection throughout sun exposure. UVA filters, on the other hand, are uncommon and insufficiently photostable. In this context, Eric Chatelain et al. used bis-ethylhexyloxyphenol methoxyphenyl triazine (Tinosorb S) for the photostabilization of AVOB and ethylhexylmethoxycinnamate. Tinosorb S was used with AVOB in the formulation and evaluated for its stability by irradiation with a xenon arc source. Chromatographic and spectroscopic techniques were used to determine the sun protection factor (SPF) and UVA ratio to count the amount of UV filters that were retrieved after being exposed to radiation. As stated by the author, Tinosorb S prevented the photocatalytic degradation of AVOB in a concentration-dependent manner, maintaining a constant SPF to UVA ratio even after exposure to radiation with intensity as high as 30 MED. Investigators tested the impact of Tinosorb S in sunscreens with this UV filter mixture because it has been found that AVB destabilizes ethylhexyl methoxycinnamate (EHM). In this study, Tinosorb S demonstrated photoprotective qualities against both UV filters. Thus, to increase the photostability and effectiveness of sunscreens that contain AVOB and EHM, Tinosorb S can be used [48].

#### 7.2.4. Nanoparticles as a Photostabilizer for Avobenzone

**Figure 4:**
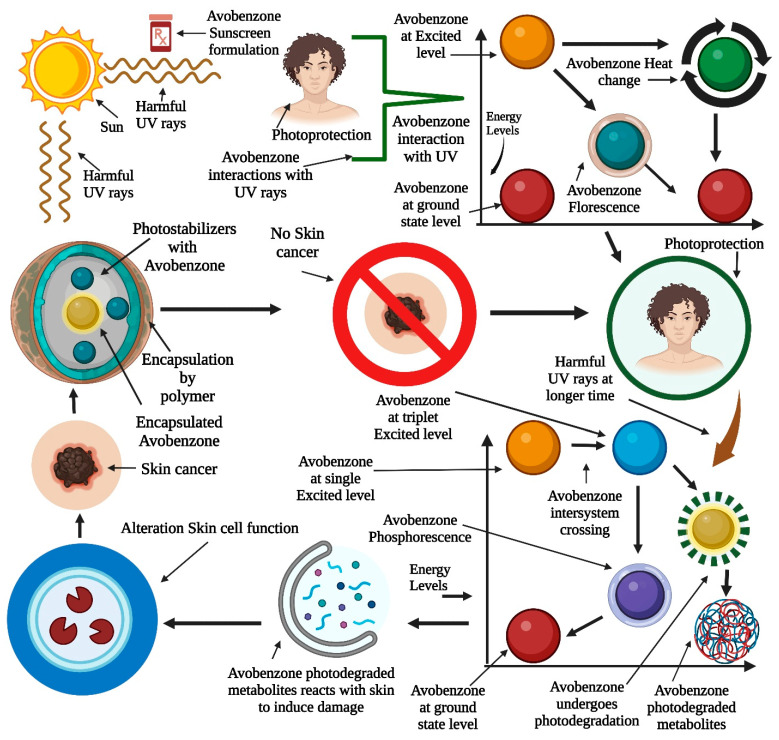
Schematic representation of sunscreen agent-loaded micro/nanocarriers wherein the delivery systems that surround the sunscreen agent prevent skin penetration and limit exposure to possibly dangerous substances. Encapsulation and photostabilizer compounds are used to make photostable formulations for photounstable UV filters such as AVOB. Generally, a stable UV filter after UV ray exposure undergoes an excited state and returns to a stable ground state by using phosphorescence and fluorescence. After prolonged UV exposure, such UV filters undergo interstate system crossing to form photograded metabolites, which further induce skin cancer.

To take advantage of the benefits of nanoengineering, nanotechnology is being included in conventional skin-care products. Carrier (polymeric NPs, SLNs, microparticles, reservoir systems, and liposomes)-based sunscreens outperform conventional sunscreens in four ways [49]: first, they are effective UV filters that can block and absorb UV radiation; second, they pack firmly on the skin for a uniform coating; third, because they are so small, they disappear into the skin, leaving no noticeable residue; and fourth, they can be combined in oil-free preparations for elegant cosmetic purposes (Figure 4).

The effect of quercetin on the photostability of AVOB was investigated by Scalia et al. [50]. They have demonstrated that quercetin was highly effective when used at lower concentrations than commonly used stabilizers (octocrylene) and antioxidants (vitamin E).

Elisa C. Felippim et al. fabricated a nanoencapsulation of a quercetin photoprotective formulation. Harmful reactive oxygen species are generated in the skin and damage skin cells due to harmful UV radiation. Authors use quercetin as an alternative antioxidant in sunscreens, which provides additional skin photoprotection. Quercetin has stability and permeation problems, so it is encapsulated in nanoparticles to improve stability and skin permeation. They prepared free and nanoencapsulated quercetin and evaluated its physical, mechanical, and sensorial properties. They also paid attention to the effects of nanocarriers on sun protection factors (SPF) and their immediate clinical effects. To evaluate immediate clinical effects and sensorial properties, they used 20- and 35-year-old female participants and biophysical and skin imaging techniques. The developed formulation shows an increase in SPF in vivo without an increase in UV filters. The created photoprotective compound enhanced skin hydration and function while having a synergistic effect on SPF. In conclusion, encapsulation technology is important for unstable photoprotective chemicals [51].

#### 7.2.5. Octocrylene as a Photocatalizer through Silica Sol-Gel Capsules

Manufacturers in this international sector are financially incentivized to design new photoprotective filters and novel technology to create the most efficient, versatile, and long-lasting sunscreen products.

In fact, sunscreens must shield against skin damage while also being safe for the skin, comfortable to the customer’s senses, and attractive in appearance. The examination of the absorption of sunscreen following topical application has been taken into account by European authorities in recent years. In this context, Arianna C. Cozzi and colleagues compared the performance of sunscreens with free and incorporated UV filters in terms of skin penetration, retention, and photostability. AVOB and octocrylene, two UV filters, were chosen and encapsulated in silica sol-gel capsules. They employed the tape stripping method to test penetration. Encapsulated UV filters last longer on the skin than free UV filters, lowering penetration. The encapsulated formulation has better photostability and a higher sun protection factor than the free form. Encapsulation technology can potentially increase the efficacy of sunscreens containing organic UV filters while lowering the risk of side effects [52].

A good photoprotective agent has properties such as maximum UV absorption, high photostability, and water resistance. To investigate the effects of encapsulation, two of the most regularly used sunscreen compounds, AVOB and OMC, were encapsulated in lipid microparticles (LMs) and analyzed. Valentina Trotta et al. fabricated lipid microparticle encapsulation of AVOB and octyl methoxycinnamate (OMC) to enhance their photostability and water resistance. The photostability test was performed by irradiating the formulation with a solar simulator. The results show a significant decrease in the photodecomposition of encapsulated UV filters compared to free UV filters. In vitro, water-resistance tests revealed that entrapment in the LMs considerably increased the amount of sunscreen agent removed by watering compared to nonencapsulated sunscreen agents. The sun protection factor shows no significant difference between free and encapsulated UV filters [53].

In another work, Santo Scalia et al. investigated how quercetin affected the photostability of several UV filter compositions. The researchers intended to see how the herbal antioxidant quercetin affects the photostability of butyl methoxydibenzoylmethane (BMDBM) and OMC. Creams containing BMDBM (3% *w*/*w*) and OMC (4% *w*/*w*) were exposed to solar radiation at an intensity similar to natural sunlight to imitate the conditions found in commercial sunscreen lotions. Quercetin helps to improve the stability of the added UV filters. The photodegradation of BMDBM and OMC was significantly reduced when quercetin was added to the sunscreen formulation. Furthermore, comparative photodegradation experiments revealed that quercetin was far more effective than routinely used stabilizers (OC) and antioxidants at lower concentrations (vitamin E, butylated hydroxyanisole). The quercetin-based formulation’s UVB and UVA protection characteristics were determined in vitro to meet the official standards for sunscreen products. Quercetin is a valuable addition to the manufacture of effective broad-spectrum sunscreens comprising BMDBM and OMC because of its photostabilization and numerous antioxidant capabilities. According to the findings of this study, including small amounts of quercetin in sunscreen formulations is an easy and productive way to improve the photostability of BMDBM and OMC [50].

Free and encapsulated UV filter comparative studies have been performed by some researchers. Arianna C. Cozzi et al. studied the comparative performance of photoprotective formulations with free and encapsulated UV filters regarding various parameters, such as skin penetration, retention, and photostability. The financial stakes for manufacturers in this industry are to invent different UV filters and novel technologies to give the most efficient, versatile, and durable photoprotective products. AVOB and OC were encapsulated in a sol-gel silica capsule. For the penetration study, they used the tape-stripping method. Compared to free UV filters, encapsulated UV filters retain skin, i.e., decrease penetrability. The encapsulated formulation shows more photostability and an improved sun protection factor compared to the free form. An encapsulation strategy is an effective approach for increasing the efficacy of sunscreen products containing UV filters while reducing the risk of side effects [54].

### 7.3. Encapsulation for Avobenzone Photostabilizer

**Figure 5:**
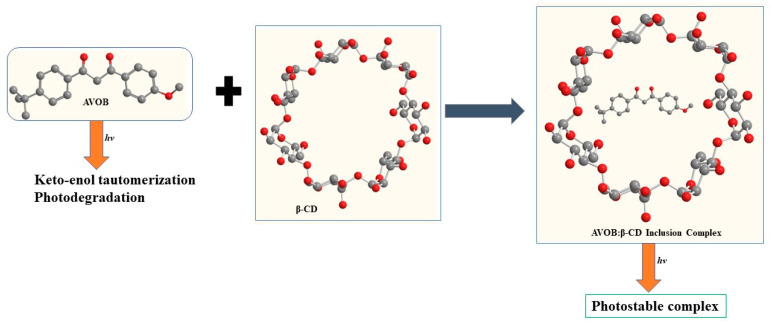
Avobenzone & β Cyclodextrin Inclusion Complex. AVOB undergoes photodegradation through keto-enol tautomerism to produce photodegraded products, which further induces phototoxicity. Such issues are countered by effective encapsulation by *β*-cyclodextrin by forming an avobenzone photostable comp.

Various novel approaches have been used to enhance the photostability of AVOB by using several nanocarriers, as reported in different studies. To address AVOB’s photoinstability, a liposome nanocarrier system through SLNs was developed and provided better results. In this context, Jing Yang et al. fabricated a hydroxypropyl-*β*-cyclodextrin (HPCD) complex with AVOB to observe the effect of HPCD on the penetration and photostability of AVOB. (Figure 5.) The modified diffusion cell apparatus used for the transdermal permeation study explained that if there is a high concentration of HPCD 30% (*w*/*w*), then there is less transdermal penetration.

AVOB was completely solubilized in cyclodextrin when flux maxima occurred at 20% HPCD. The AVOB reservoir was formed on the skin upon reaching 30% HPCD, resulting in a decrease in transdermal penetration and enhancing the photoprotective effect. The most stable formulation was found to be 30% (*w*/*w*) HPCD. An in vivo study showed that less edema induction and sunburn cell formation by the 30% (*w*/*w*) HPCD formulation was considered the best photoprotective formulation. This work showed that there is less skin penetration and photodecomposition of AVOB with enhanced photoprotection activity through cyclodextrin encapsulation [55].

In another work, Santo Scalia et al. fabricated a complex of butyl-methyl benzoyl methane and hydroxypropyl-cyclodextrin in the liposphere. This study explained that the complex containing hydroxypropyl cyclodextrin (HPCD) and the UV filter butyl methoxydibenzoylmethane (BMDBM) inserted into lipospheres affected the photostability of sunscreen. For the preparation of lipid microparticles, they used hydrogenated phosphatidylcholine and tristearin as emulsifiers and lipids, respectively. When BMDBM was integrated as an inclusion complex compared to a free molecule, the release of BMDBM from the lipospheres was delayed. Sunscreen-loaded lipospheres, BMDBM/HPCD complex lipoparticles, and their combination with HPCD were all irradiated and incorporated into a model cream (oil-in-water emulsion). According to the photodegradation tests, all of the tested systems significantly reduced the amount of light-induced breakdown of the free sunscreen material (BMDBM loss fell from 28.9% to 17.3–15.2%). Photocatalytic experiments over three months revealed that the photoprotective characteristics of the complex remain unchanged and have high stability [56].

#### 7.3.1. Encapsulation Using β-Cyclodextrin and Its Derivatives

The skin penetration along with skin permeation study for AVOB was performed by some researchers to report further retention of AVOB into the skin after topical application through sunscreen formulations. S. Scalia et al. fabricated AVOB with hydroxypropyl-*β*-cyclodextrin (HP-CD) and sulfobutylether (7) *β*-cyclodextrin (SBE7-CD) to study the skin penetration and retention of AVOB, which is a sunscreen agent that absorbs UVA radiation. The AVOB and cyclodextrin interaction was understood in water by using phase solubility analysis. There was no interaction between AVOB, and the cyclodextrin co-evaporation method was used to prepare the solid complexes. Franz diffusion cells were used to conduct in vitro human skin penetration research. AVOB-free or AVOB-complexed solutions were placed on excised skin in Franz diffusion cells. HPLC was used to determine how much sunscreen permeated into the stratum corneum, viable epidermis, dermis, and receptor fluid after 6 h. The results show that in skin tissue, 14.10–16.78% of the applied dose penetrated, and there was no agent found in the dermis and receiver phase. A total of 84.6–95.5% complex and uncomplex AVOB was found in the stratum corneum. Free AVOB (2.29% of the applied dose) was found in the epidermis, and when complexed with SBE7-CD, only 0.66% of the applied dose was found in the epidermis, and no noticeable effect was observed with the HP-CD complex. Therefore, this complexation limits the direct contact of the UV filter with the skin [57].

In another work, Simone d’Agostino et al. formulated a cyclodextrin inclusion complex of AVOB and octinoxate to improve photostability and formulate environmentally friendly sunscreens. The author of this study employs AVOB and octinoxate (OC), two commonly used sunscreens. Solid-state methods such as XRD, Raman, and ATR-FTIR spectroscopy were utilized to confirm the development of crystalline inclusion complexes. The researchers identified and characterized a novel metastable polymorph of AVOB. The presence of sunscreen compounds in the hydrophobic cavity of *β*-cyclodextrin was assessed using mass spectrometry (ESI-MS) and UV–VIS spectroscopy for photodegradation experiments in solution. The photodegradation of sunscreen compounds was shown to be significantly reduced. The incorporation of AVOB and OC in *β*-cyclodextrin increases photostability, according to the author, and this is a potential route for the development of ecologically friendly sunscreens [58].

Several studies have shown that inclusion complexes embedded with polymers seem to be helpful for photostability for sunscreen formulation issues. Layan Dahabra et al. fabricated a cyclodextrin inclusion complex containing sunscreen to prevent skin diseases. UV radiation (UVR) damages the DNA of skin cells, leading to skin cancer. To protect the skin from UVR, sunscreen formulations contain UV-protecting agents that absorb, scatter, or reflect UVR. Natural antioxidants found in sunscreen formulations help to stabilize them during and after manufacturing. After exposure to sunlight, these agents start the degradation process. To prevent degradation, UV filters are incorporated into the cyclodextrin complex. According to the research, incorporating antioxidants and UV filter components into the CD cavity is a viable way to increase sunscreen efficacy. With the help of these CDs, antioxidants and UV filters are more stable when exposed to light and oxygen. Additionally, CDs can significantly increase the bioactivities of hydrophobic antioxidants, particularly as anticancer agents, and decrease the percutaneous absorption of sunscreen compounds [59].

Some researchers have implemented modified inclusion complexes by using dextrin to address skin penetration issues of AVOB, which has exhibited vital results. Chia-Ching Li et al. applied encapsulation technology for the preparation of AVOB encapsulation in modified dextrin to reduce skin penetration and improve UV protection. DSC, TGA, and UV spectrophotometry were used to examine the encapsulation efficiency and characteristics of the organic UV filter inclusion complex. Different evaluation parameters of AVOB with or without encapsulation in modified dextrins, such as dissolution, release, in vitro sun protection factor, and skin penetration study, were also examined. After UV irradiation, there are no traces of normal photoreaction byproducts. Finally, the photostability of this compound was confirmed by UV-VIS spectroscopy. From this research, they demonstrated that a viable path for the creation of enhanced sunscreens and novel sunscreen formulations is possible, with a focus on environmentally friendly products. By improving the photostability of the AVOB and octinoxate inclusion complexes in cyclodextrin, sun creams can also use fewer UV filters, which is good for both the skin and the environment [60].

#### 7.3.2. Liposphere Drug Delivery System

To limit the photodegradation of UV filters induced by sunlight, the liposphere was formulated to provide effective encapsulation and photostability of UV filters. In this study, Iannuccelli et al. fabricated a liposphere containing AVOB with the objective of understanding skin penetrability. The skin penetration of lipid microparticles loaded with AVOB, a commonly used UV-A sunscreen chemical, synthesized by the melting approach and the tested formulation of AVOB incorporated in the liposphere resulted in 15% of the applied sunscreen accumulating in the stratum corneum without affecting the skin penetration of unencapsulated sunscreen. Using a synthetic lipophilic membrane, AVOB diffused into a hydrophilic phase, which was able to predict these results better than using an ethanolic phase. This strategy reduces the decomposition and skin penetration of BMDBM. These are suitable components for sunscreen agents to increase the expected UVA protective capacity and safety [61].

#### 7.3.3. Morin-Encapsulated Nanoparticles

Natural flavonoids such as morin have excellent antioxidant properties and are used for the photostability of AVOB by implementing dual photoprotection through encapsulating polymers and antioxidant agents. The drug delivery system for the same is based on nanocarriers, which will help to reduce further penetration of UV filters through the skin. Pallavi Krishna Shetty et al. developed morin-encapsulated nanoparticles (NPs) to increase the protection from UV radiation and the antioxidant activity of sunscreen creams. They prepared and optimized morin-containing polymeric nanoparticles and further prepared and evaluated creams containing morin nanoparticles. The particle size of the optimized nanoparticles was 90.6 nm, and the zeta potential was −31 mV. Morin was found to be entrapped by polymeric nanoparticles in only 12.27% of cases, which is very low. Using Fourier transform infrared spectroscopy (FTIR) and differential calorimetry (DSC), it was concluded that morin and excipients did not interact. The author discovered that the nanoparticles were spherical with a diameter of approximately 100 nm. The optimized NP in vitro free radical scavenging performance was exceptional. Its NPs had stronger skin penetration and morin deposition than the simple version. Several sunscreen creams (SC1–SC8) were created. The SC5 and SC8 creams had excellent sun protection factors (SPF; 40). Sunscreen lotions containing morin NPs were found to have excellent skin penetration in vitro and in vivo skin penetration studies. A study in Vero and HaCaT cells did not show cytotoxicity of morin nanoparticles and cream formulations (SC5 and SC8). Sunscreen lotions with better cutaneous protection have been discovered. The SC5 and SC8 creams demonstrated remarkable in vivo antioxidant activity in UV-exposed rats. The improved sunscreen creams demonstrated excellent UV protection and antioxidant capabilities [62].

#### 7.3.4. Encapsulation by Polymethyl Methacrylate

The effective photostabilization of the UV filter is regulated by encapsulation. Effective encapsulation is dependent on drug entrapment and polymer selection. Several nanoparticles using different polymers were used for AVOB photostabilization. Pey-Shiuan Wu et al. investigated the ultraviolet radiation absorbance and, in vitro, sun protection factor (SPF) of novel polymethyl methacrylate (PMMA)-encapsulated organic ultraviolet (UV) filters. The selected polymer provided effective entrapment of the drug and facilitated the application of nonbiodegradable polymers to the skin. Sunscreen formulations have prevented the skin from many damaging effects of ultraviolet (UV) light, so their stability is a primary consideration. As a result, discovering novel UV filters or modifying existing UV filters is a crucial step in sunscreen design. In this study, researchers introduced encapsulated benzophenone-3 (TB-MS), AVOB (TA-MS), TO-MS, diethylamino hydroxy benzoyl hexyl benzoate (TD-MS), and other unique poly(methyl methacrylate) (PMMA)-encapsulated organic UV filters. (UV filters encased in PMMA have improved photoprotection, photostability, and safety.) As a result, the author speculates that these PMMA-encapsulated UV filters could be used as photoprotective components [63].

#### 7.3.5. Avobenzone Solid Lipid Microparticles (SLMs) by the Spray Congealing Technique

The SLMs provide better drug loading capacity for hydrophobic drug molecules. They are also known as good drug carriers for the effective chemical stability of encapsulated compounds. To address phototoxic and photoallergic reactions induced by photounstable AVOB, the application of SLMs is very useful. To justify this, Beatrice Albertini et al. fabricated solid lipid microparticles (SLMs) by a spray congealing technique using the UVA absorber AVOB to reduce photoinstability. The spray congealing technique was used for the preparation of microparticles. The entrapment efficacy of sunscreen agents was 40.1 to 48.5% (*w*/*w*). AVOB release from SLMs prepared by the spray congealing technique is more efficient. A photodegradation study indicated that nonencapsulated AVOB degrades at 38.6% ± 3.6 and 15.4 ± 4.1% for microparticle-entrapped sunscreen prepared by the spray congealing technique. For fast and free solvent preparation of SLMs, the spray congealing technique outperforms the classical melt dispersion method regarding entrapment efficacy and photodegradation. Additional benefits of the spray congealing approach include inexpensive operating costs, high adaptability, and the ability to make SLMs without using surfactants or solvents. The congealing approach for lipid microparticle generation is a promising technology for future sunscreen formulation development [64].

In another work, Santo Scalia et al. fabricated solid lipid microparticles of butyl methoxybenzoylmethane with a photo stabilizer to increase the photostability of UV filters. One of the most extensively used UV-A filters, butyl methoxydibenzoylmethane (BMDBM), decomposes in sunlight. This work examines the incorporation of BMDBM and the photostabilization of 4-methyl benzylidene camphor (MBC) into solid lipid microparticles (SLMs) to minimize sunscreen photodegradation. The melt dispersion procedure was utilized to create microparticles from several lipid components with hydrogenated phosphatidylcholine as the surfactant. The tristearin microparticles had the best BMDBM and MBC retention capability. BMDBM and MBC had loadings of 10.4% and 10.1%, respectively. After being put into a conventional cream, the efficacy of the SLMs was assessed (oil-in-water emulsion). The light-induced breakdown was minimized by encapsulating BMDBM into SLMs. Furthermore, when the MBC stabilizer was coloaded in the SLMs, the UV-A filter photodegradation decreased much more (BMDBM loss was 16.9 ± 5.9%) compared to microparticles containing BMDBM without MBC. As a result, combining BMDBM with the MBC photostabilizer in lipid microparticles improves UV-A filter photostability more effectively than SLMs loaded with BMDBM alone. The encapsulation technique should limit the negative interactions of the UV-A filter with the skin, lowering the potential toxicological concerns [65].

#### 7.3.6. Solid Lipid Nanoparticles (SLNs) and Nanostructured Lipid Carriers (NLCs)

SLNs and NLCs were developed by Xia et al. as UV carriers. Lipid NPs produced with a solid matrix were used to provide UV protection. Molecular sunscreens have been claimed to be capable of loading between 10 and 15%. With this novel UV protection technique, it was possible to load up to 70% of molecular sunscreen onto NLCs, which is enough to produce high SPFs. The suggested sunscreen formulas offer a viable substitute for conventional sunscreen formulas. The lipid particles’ UV protection efficiency varied depending on the type of lipid and the UV wavelength. The UV-blocking impact is improved by incorporating sunscreens into lipid carriers [41].

The author develops lipid nanoparticles that contain butyl methoxydibenzoyl methane (BMDBM). All the required evaluation parameters of lipid nanoparticles (LNs) were tested. The efficacy of lipid nanoparticles in the production of numerous cosmetic formulations was evaluated using the in vitro erythemal UVA protection factor. To assess the photoprotective effect, various cream formulations based on BMDBM-LNs and a standard emulsion were subjected to low-intensity UV rays to simulate the blazing sun. The results showed a high capacity for UVA absorption of more than 96% in cream formulations based on BMDBM-LNs. Furthermore, the developed cosmetic formulations have a fourfold higher erythemal UVA protection factor than standard emulsions, indicating a better UVA-blocking activity [66].

To investigate the photostability, photoprotection, and in vitro release of lipid NPs containing butyl-methoxydibenzoylmethane and OC, Gabriela Niculae et al. produced lipid nanoparticles containing butyl-methoxydibenzoylmethane and OC. Scientists combined the UV filters butylmethoxydibenzoylmethane (BMDBM) and OC to generate stable sunscreen formulations with lipid nanocarriers. Several nanocarriers were examined in various concentrations of the two UV filters to determine which combination of absorption and release qualities worked best. The two forms of lipid nanocarriers used are SLNs and NLCs. The creams with low UV filter levels (2.5% BMDBM and 1% OC) contained nanocarriers. Cream-based MCT NLCs had the best photoprotection efficacy, with a sun protection factor (SPF) of 17.2 and an erythemal UVA protection factor (EUVA-PF) of 50.8. The photostability of the incorporated BMDBM filter was proven by in vitro irradiation of nanocarrier-based creams. The synthetic UV filters are released slowly in vitro, following the Higuchi release model, resulting in sustained UV protection efficacy. This work’s results show that the encapsulation of BMDBM and OC into lipid nanocarriers was accomplished using the high-shear homogenization approach. All of the formulations showed the desired results of all parameters and stability. As a result, a commercial broad-spectrum sunscreen with significantly lower UV filter concentrations and better UV protection might be developed compared to standard formulations with the same number of UV filters. Furthermore, an in vitro release investigation revealed that encapsulating UV filters in lipid nanocarriers reduces skin penetration, resulting in a high level of safety [67].

Solid lipid nanoparticles (SLNs) are considered the best conveyer to enhance sunscreen protection and efficacy. The impact of the type of surfactant on the stability of encapsulated UV filters in SLNs was investigated. Montenegro et al. fabricated solid lipid nanoparticles containing sunscreens by the phase inversion temperature method. Butylmethoxydibenzoylmethane (BMBM) and OMC UV filters were used to study the effect of surfactant on UV filters, and differential scanning calorimetry (DSC) was used to study the interactivity between solid lipid nanoparticle components and loaded UV filters. Using isoceteth-20 or oleth-20 as the main surfactant did not result in the expected physiochemical characteristics. The OMC was dispersed within the solid lipid nanoparticles and caused a reduction in the cooperativity of the lipid substrate molecules, according to DSC analysis, whereas the BMBM did not affect the SLN’s calorimetric activity. A reaction between the BMBM and the OMC occurred when the OMC and the BMBM were incorporated into these SLNs concurrently. These findings indicate that reactions between sunscreen and solid lipid nanoparticle components should be studied further to assess their effect on the efficacy of UV-containing SLNs. Major surfactants, such as isoceteth-20 or oleth-20, did not offer suitable SLNs to load OMCs and BMBMs when cetyl palmitate was utilized as a solid lipid to prepare SLNs, according to the scientists’ findings. The SLN loaded with the resulting UV filter is stable when ceteth20 is used as the major surfactant, but BMBM loading occurs. The prospects for more research into the consequences of such interactions between OMCs and BMBMs are bleak. According to the DSC analysis, the OMC incorporated inside the solid lipid nanoparticle caused a reduction in the cooperativity of the lipid substrate molecules. Nevertheless, there was no change in the calorimetric behavior of the SLNs after loading the BMBMs. Furthermore, an interaction between the BMBM and the OMC is released from the SLNs when the OMC and the BMBM are loaded together into these SLNs [68].

L. Coelho et al. [29] outlined different strategies for the photostabilization of photosensitive drugs were studied. Degradation or photoinstability results in a loss of biological activity. Researchers have explored whether the encapsulation strategy has great potential for the photostabilization of photosensitive drugs. Cyclodextrin (CD) was the most commonly utilized encapsulation technology (32.5%), followed by liposomes and lipid nanoparticles (17.5%), microparticles (15%), and polymeric nanoparticles (15%). Liposomes and nanoemulsions contribute equally due to their phospholipid bilayer and hydrophilic and hydrophobic cores.

A liposome and lipid nanoparticle strategy proved the most successful. Choosing a photostabilization strategy involves researching the photodegradation mechanism, assessing the final product’s effectiveness of photostabilization and comparing alternative strategies to look for the best one from a cost-benefit perspective. Finally, assurance of the pharmaceutical product’s efficacy should be highlighted. Future studies in this area should examine the photostability of compounds in solution vs. when they are incorporated into a formulation. In the future, photostability experiments performed with a solar radiation exposure environment may become more valuable, allowing for easier comparisons. Understanding photodegradation pathways is a crucial step in the design of pharmaceuticals [69].

In another work, D. Nesseem et al. produced sunscreens with an increased UV protection factor using solid lipid nanoparticles. The goal of this study is to design and develop sunscreen carrier systems based on a decyl oleate and carnauba wax matrix. To generate formulas (F1–F7), butyl methoxydibenzoylmethane and OMC were used as organic components, and titanium dioxide (TiO_2_) was used as an inorganic component. Using the usual method of production, both types of sunscreens are combined to create SLN formulations. To determine how the colors influenced the nanoparticles, a Mastersizer particle size analyzer was used to assess particle size. The in vitro SPF test was used to assess the UV-protection capacities of the formulations. The spreadability and viscosity of the fluid were also determined. The rheological behavior of the formulations was also investigated. Significant increases in SPF values of up to 50 were seen after encapsulation with organic and inorganic filters in Canada wax and decyl oleate. As a result, both organic and inorganic sunscreens might be transported through SLNs. Cinnamates, titanium dioxide, and zinc oxide, when used together, have been shown to increase the SPF of cosmetic preparations in a synergistic way. The intrinsic SPF of the studied inorganic compounds was increased when they were encapsulated. The viscosity of the systems was positively modified by the mixed lipid matrix used, allowing the formulations a better fixation after being applied to plates for SPF assessment [70].

#### 7.3.7. Avobenzone Coating with Mesoporous Silica Particles (MPSs)

The synthesized silica has a high specific surface area along with excellent photostability. These materials provide less cellular toxicity and can be considered an effective tool as carriers for the delivery of photounstable UV filters. Tai Yong Lee et al. developed and tested AVOB coated with mesoporous silica particles (MPSs). Mesoporous silica particles are generally prepared by using surfactants followed by condensation. Photostability was tested under sun-simulated light; the effect on UV protection qualities was evaluated. Surprisingly, the composition of the particles has a substantial impact on UV protection. The UV blocking characteristics of materials with SPF values ranging from 4.61 to 11.81 were improved by encapsulating AVOB and benzene-containing organosilica coatings. In addition, the in vivo sunburn protection properties of nude mouse skin were investigated. The thickness of the epidermis was less noticeable in the skin tissue treated with materials in particular. Surprisingly, although it was 11 times thinner than the unprotected skin, the epithelium of the skin tissue exposed to the material did not thicken. The materials exhibit significant photodegradation stability as well as the considerable potential for usage as UV protection agents. Organosilica coating layers enhanced the UV-blocking abilities of materials, and UV absorbing performance was improved by encapsulated AVOB [71].

#### 7.3.8. Microencapsulation of Butyl Methoxydibenzoylmethane

Santo Scalia et al. fabricated butyl methoxydibenzoylmethane microencapsulated in cyclodextrin. The effect of lipid microparticles containing a combination of hydroxypropyl cyclodextrin (HPCD) and the sunscreen compound butyl methoxydibenzoylmethane (BMDBM) on UV filter percutaneous penetration was investigated. Melt emulsification was used to make the microparticles, with tristearin as the lipidic substance and hydrogenate phosphatidylcholine as the surfactant. The tape-stripping technique was used to examine human skin penetration in vivo. After solvent extraction, HPLC was used to determine the amount of sunscreen fixed to each strip. The UV filter was recovered at a rate of >94.4% from spiked adhesive tape, and the method’s precision was greater than 7.6% relative standard deviation. The encapsulated and nonencapsulated formulations were given to human participants, and the results were compared, increasing the amount of BMDBM diffusing into the stratum corneum (percentage of the applied dose penetrated, 9.7%). The cream containing the microencapsulated BMDBM/HP-CD complex, on the other hand, drastically reduced the quantity of UV filter that reached the stratum corneum (percentage of the administered dose penetrated, 6.0% 1.5%). The decreased BMDBM percutaneous penetration of the latter technology should improve UV filter efficacy while limiting potential toxicological consequences. The findings of this study indicated that the tape-stripping approach might be used to infiltrate BMDBM into the skin. Furthermore, adding BMDBM as an HP-CD complex to lipid microparticles inhibited sunscreen penetration into the stratum corneum. Because the fraction that reaches deeper viable skin tissues and the systemic circulation is proportional to the concentration in the stratum corneum, the results demonstrated that lipoparticles loaded with complex BMDBM hindered the sunscreen agent’s percutaneous absorption. The effect not only increases the protective capacity of the UVA filter by keeping it at the skin’s surface but also reduces the risk of harmful reactions [72].

#### 7.3.9. Mesoporous Silica Encapsulating Avobenzone

Wei-Hsun Wang et al. prepared hierarchically mesoporous silica encapsulating AVOB. This hierarchically mesoporous silica (HMS) has good photostability and no cellular toxicity. Synthesized silica is an excellent candidate for a carrier material. Based on nitrogen adsorption and desorption studies, the structure of the hysteresis loop confirmed the presence of mesoporous pores in the HMS powder. In addition, the encapsulation efficiency was higher than 90%. AVOB was encapsulated in the HMS powder due to its large specific surface area and pore volume. Furthermore, the new synthetic sunscreen maintained its remarkable UVA absorption capabilities after being encapsulated. The large specific surface area resulted in mesoporous materials. The rate of reduction in the specific surface area was 95.12%, and the rate of reduction in the pore volume was 91.27%. MSAB stands for AVOB encapsulated powder. To analyze HMS before and after AVOB encapsulation, the results of nitrogen adsorption-desorption studies were calculated. Furthermore, the AVOB characteristic bands in MSAB did not change, indicating that no chemical connection was formed between HMS and AVOB. The ratio of HMS to AVOB in MSAB is 2:1 according to TGA curves. The result is the same as the initial HMS/AVOB ratio. This demonstrated that the HMS powders with AVOB encapsulation still have excellent UV protection [73].

#### 7.3.10. Mesoporous Silica SBA-15 and UV Filters

To minimize the skin penetration of organic UV filters, Andre Luis Maximo Daneluti et al. employed mesoporous silica SBA-15. AVOB (AVOB), oxybenzone (OXY), and OMC are some of the most regularly used UV filters [74]. In this investigation, the researchers wanted to explore how different types of mesoporous silica (SBA-15) affected cutaneous deposition and permeability. Stick formulations with “free” and “integrated” UV filters (SF1 and SF2, respectively) were evaluated. As assessed by UHPLC-MS/MS, skin deposition of AVOB and OXY following treatment with SF2 for 6 and 12 h was considerably lower than that after treatment with SF1 at each time point (Student’s *t*-test, e.g., after 6 h, 12 h, and 24 h after applying SF2, OXY permeability across the skin was checked. A considerable drop in the viable epidermis and dermis quantities was seen in the cutaneous biodistribution profiles of AVOB and OXY. Deposition of the more lipophilic OMC, on the other hand, was not substantially different (*p* ˃ 0.05) [75].

Adsorption/entrapment of UV filters increased the sun protection factor by 94% in in vitro photoprotective efficacy tests. Finally, SBA-15, a novel mesoporous polymer, improved UV filter photoprotection while lowering cutaneous penetration and transdermal permeability. UV filters were entrapped inside SBA-15 and adsorbed on the mesoporous material’s surface; a formulation containing AVOB, OMC, and OXY was successfully developed and reported. When UV filters were added to the stick formulations, the SPF increased by 90% when compared to “free” UV filter controls. Skin permeation and deposition investigations demonstrated that the stick formulation with “integrated” UV filters drastically reduced the transdermal penetration of OXY and cutaneous deposition of OXY and AVOB when compared to the nonincluded stick formulation. SBA-15 lowered the levels of OXY and AVOB in the stratum corneum, viable epidermis, and dermis after 6 h and 12 h of treatment, according to the biodistribution data. Both formulations delivered OMC with no significant difference. OMC is expected to be more soluble in the formulation’s lipophilic components, allowing it to stay in the formulation longer. According to the findings, embedding UV filters into SBA-15, a unique and safe nanostructured material, might reduce UV filter cutaneous penetration and transdermal permeation, resulting in decreased systemic exposure and increased photoprotection [71].

### 7.4. Nanostructured Lipid Carriers (NLCs) and Nanoemulsions (NNs)

To enhance the accumulation of added UV filters in sunscreen formulations at the administration site along with excellent water resistance, several nanocarrier approaches, such as lipid nanoparticles and nanoemulsions, were used. These systems will also boost SPF via extended UV filter photostabilization. Carmelo Puglia et al. fabricated NLCs and NEs in which UV filters are incorporated to increase their photostability and reduce skin penetration. UVA or UVB sun filters were used in this study, such as ethyl hexyltriazone (EHT), diethylamino hydroxy benzoyl hexyl benzoate (DHHB), bemotrizinol (Tinosorb S), OMC, and AVOB. UV filter carriers are prepared, and their stability is studied by considering particle size and zeta potential. After the successful preparation of UV carriers, UVA and UVB agents are incorporated into them and evaluated for different in vitro parameters, such as photostability and skin permeability. *The* in vitro skin permeation ability performed on excised human skin shows that the skin permeation ability is drastically reduced once UV filters are incorporated into NLCs. Incorporated EHT, DHHB, and Tinosorb S in UV carriers show photostability after irradiation, while AVOB and OMC do not show the expected photostability. Both carriers showed better photoprotective efficacy. From this study, it is concluded that the nanostructure lipid carrier area is a significant strategy for reducing skin penetration ability and increasing the photostability of UV filters [76].

### 7.5. Titanium Dioxide Coating for Avobenzone Photostabilization

One of the widely used physical barriers for UV filters in sunscreen formulations is TiO_2_. The implemented physical UV filters, such as TiO_2_, vary their photoprotection for photosensitive drugs due to skin whitening issues. These issues are induced by TiO_2,_ as they scatter visible light and need better modification for their effective utilization. Can Wang et al. fabricated a broad-spectrum sunscreen called TiO_2_-AVOB by coating AVOB on prepared TiO_2_. The TiO_2_ surface was encapsulated by the organic film layer to form the core-shell structure. The formulation was made with TiO_2_-AVOB and then tested using a droplet size distribution analyzer, HPLC, and an ultraviolet transmission analyzer. TiO_2_-AVOB had superior dispersibility, a decreased degradation rate, and an increased sun protection factor value when compared to individual components in the produced emulsion, implying that TiO_2-_AVOB might be used as a sunscreen contender [77].

#### Micronized Titanium Dioxide (TiO_2_)

In cosmetic formulas, there is a demand for ideal sun protection that is acceptable, effective, and safe. Aesthetics demand that the product be simple to apply and spread evenly across the skin’s surface, as well as virtually unnoticeable to the eye. By scavenging reactive oxygen species, photostable materials coupled with antioxidants can provide good sun protection. The UV-absorbing or UV-reflecting material can be left on the surface of the stratum corneum, which provides a barrier from the sun’s damaging rays. The author created a method that combines antioxidants with micronized titanium dioxide (TiO_2_) to create UV—visible transparent polymer gel particles. White light backscattering is reduced by the micronized TiO_2_ particles, which are coated with silica to avoid clumping. UV radiation causes oxidative stress in gel-trapped TiO_2_, which boosts the photostability of some complementing chemicals, such as AVOB. The particles are in the micrometer range in size. This contributes to their long-term survival at the stratum corneum’s top. Gel-trapped TiO_2_-based sunscreens have a higher SPF and double the UVA protection of equal-composition sunscreens with more untrapped TiO_2_. Gel-trapped TiO_2_ is a better product than other photoprotective products because it stays on the skin surface, stabilizes other sunscreens, is photostable, and uses less TiO_2_ to achieve the required sun protection factor and UVA protection [78].

### 7.6. Vitamins such as A, C, and E as UV Filters for Avobenzone

**Figure 6:**
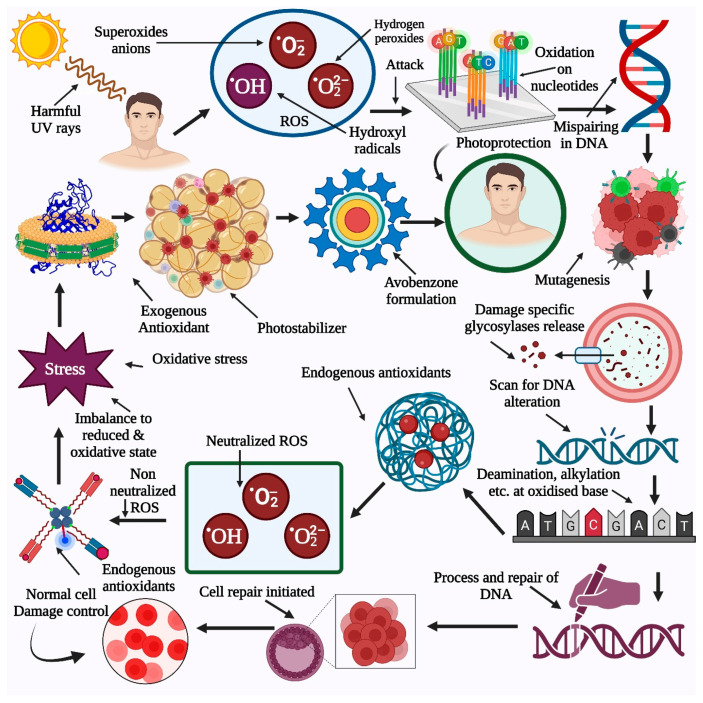
Mechanism of reactive oxygen species interactions with the body to induce skin cancer and other side effects. Several antioxidants are used to counter damage, such as DNA damage, apoptosis, and cell proliferation, induced by ROS. Different process parameters, including metabolic reactions in peroxisomes, oxidative phosphorylation in mitochondria and enzymatic reactions, play pivotal roles in the generation of ROS. Endogenous antioxidants are implemented to stabilize reactive oxygen species. In the absence of their interference, the enhanced oxidative stress will form several harmful effects on the body that need to be addressed by exogenous antioxidant agents.

Natural antioxidants are used for sunscreen formulation to reduce phototoxicity along with photoallergic reactions induced by reactive oxygen species from photodegradation. Reactive oxygen species induce DNA damage, cell proliferation and apoptosis. To limit such harmful effects, effective encapsulation and antioxidants can be implemented for photoprotection, as represented in Figure 6. Several natural antioxidants, such as vitamin C, have been tested for effective photostabilization by some researchers. Gaspar et al. fabricated photoprotective formulations of UV filters with vitamins such as A, C, and E to obtain a stable formulation. Photostability is the main factor in its use on human skin. Oftentimes, these formulations include vitamins A, C, and E. It is not yet known whether UV filters impair the ability of these vitamins to hydrate and protect the skin, especially when photostable UV filters such as AVOB are used. In this study, two UV filters, photostable and photo unstable, were combined with vitamin E, C, and A derivatives, and the influence of these filters on the photostability and efficacy of the developed formulation was observed. OMC, AVOB, and 4-methyl benzylidene camphor (MBC) make up a light-stable UV filter combination, while OMC, benzophenone-3 (BP-3), and OC make up a photostable UV filter combination (OC). Noninvasive biophysics techniques were used to understand the hydration and antiaging effects of these formulations. For this, hairless mice were used. The results demonstrate that both UV filter combinations had no effect on the formulations’ antiaging and hydration properties and that vitamins tested in surface and deeper layers reduced skin irritation. Both UV filter combinations, i.e., photostable and photounstable, were found to boost vitamin A photostability in the photostability study. However, the most photounstable formulation, which included solely vitamins, irritated hairless mouse skin and hence was not as safe as the others. The protective coating formed in the stratum corneum by these lipid-soluble UV filters, as well as the protective effects of the UV filters, were most likely responsible for the reduction in skin irritation. Finally, UV filters may be good for reducing skin irritation, and the optimal formulation includes a combination of vitamins A, C, and E, as well as photostable UV filters [79].

#### 7.6.1. Nanostructured Vegetable Oils of Sunscreens

Nanostructured vegetable oils have aided in the development of a significant change in sunscreen composition. Organic UV filters in traditional sunscreens can be absorbed into the body through the skin. As a result, prolonged contact with these chemical agents could jeopardize consumer safety and health. Furthermore, UVR can cause structural changes or degradation in organic filters such as AVOB, lowering their absorption capacity. As a result, the daily use of sun protection products is strongly advised for preventing skin-related diseases. Nanostructured vegetable oils have aided in the development of a significant change in sunscreen composition. Ultraviolet radiation (UVR) can cause structural changes and degrade organic filters such as AVOB, reducing their absorption ability. Fatty acids, polyphenols, oligo-elements, and beta-carotene are all abundant in bocaiuva oils. This feature has the potential to be used as a natural UVR defense tactic. Andre R. Babya et al. fabricated nanocarriers for *Acrocomia aculeata* oil using high-pressure homogenization technology. The developed NLCs had average diameters ranging from 106.9 1.6 to 188.4 2.2 nm, as well as a zeta potential greater than 30 mV. The AVOB entrapment efficiency (EE) of the NLCs containing bocaiuva almond oil (BAO; NLC-BAO) and bocaiuva pulp (BPO; NLC-BPO) was 75.2 and 33.3% *w*/*w*, respectively. The EE for OC was identical in both NLCs: 82.3 and 82.5% *w*/*w*. After being incorporated into a hydrophilic cream base with SPF 14, NLC-BAO was coloaded with UV filters, allowing for a two-fold improvement in the sun protection factor (SPF), from 14.1 0.7 to 31.8 0.6. Surprisingly, without AVOB or OC, NLC-BAO increased the SPF of this cream base to 27.7 0.8. As a result, NLC-BAO may be used to replace organic filters while providing photoprotection. To boost the photoprotective action of the formulation, the author effectively produced a nanocarrier incorporating bocaiuva almond oil. It has the potential to be an effective adjuvant to sunscreen products [80].

#### 7.6.2. Trans-Resveratrol and Beta Carotene for Sunscreens

J.V. Freitas et al. investigated the trans-resveratrol and beta-carotene contents of commercial sunscreen formulations. Skin penetration is a crucial consideration when applying sunscreens containing antioxidants topically [81]. This research examined the cutaneous absorption of trans-resveratrol (RES), beta-carotene, and marketed UV filters. Octyl methoxycinnamate, AVOB, and bemotrizinole-containing formulations were created and supplemented with BTC, RES, or both compounds separately or in combination, or not at all [82]. The biological membrane used in the penetration tests was porcine ear skin with Franz vertical diffusion cells. HPLC was used to assess UV filters and antioxidants in the receptor fluid, viable epidermis plus dermis, and stratum corneum (SC). The results showed that despite not penetrating the skin, UV filters and antioxidants were retained for 12 h after application. BTC, either used alone or in conjunction with RES, was observed to reduce UV-filter skin retention by 63% on average [83,84].

#### 7.6.3. Vitamin A Palmitate as a Photostabilizer

Marcela Silva Scarpin et al. studied the effects of stabilizers in AVOB and OMC on their photostability when combined with vitamin A palmitate. For cosmetic and dermatologic products, developing fresh, high-performance, and safer antiaging solutions based on new compounds to increase the stability of retinyl palmitate coupled with broad-spectrum UV filters is a problem. As a result, the goal of this research was to determine how three commonly used AVOB stabilizers, ethylhexylmethoxycrylene (EHMCR), tris (tetra-methyl hydroxypiperidinol) citrate (TTMHP), and tris-biphenyl triazine (TBPT), affected the stability and toxicity of AVOB, octyl methoxy (OMC). The formulations were exposed to UVA light for stability testing. Phototoxicity was assessed using the 3T3 neutral red uptake phototoxic test (OECD TG 432). The addition of EHMCR, TBPT, and TTMHP to the formulations boosted the photostability of AVOB and OMC in both the presence and absence of RP, although EHMCR was the most successful in stabilizing RP. The combinations of AVOB-OMC with or without RP demonstrated phototoxic capacity in the phototoxicity test. EHMCR and TTMHP both decreased the phototoxicity of the AVOB-OMC combination, while EHMCR also decreased the phototoxicity of the RP combination. As a result, EHMCR may be used to photostabilize AVOB-OMC formulations with or without RP, whereas TTMHP could be added to this photounstable UV-filter combination. The addition of EHMCR to this mixture increased the photostability of both compounds, resulting in a reduction in phototoxicity [85].

#### 7.6.4. Vitamins A, C, and E as Photostabilizers

R. Gaspar et al. fabricated photoprotective formulations of UV filters with vitamins such as A, C, and E to obtain a stable formulation. Photostability is the main factor in its use on human skin. Vitamins A, C, and E are widely used in photoprotective products. In this study, two UV filters, photostable and photounstable, were combined with vitamin A, C, and E derivatives, and the influence of these filters on the photostability and efficacy of the developed formulation was observed. OMC, AVOB, and 4-methyl benzylidene camphor (MBC) make up a photounstable UV filter combination, while OMC, benzophenone-3 (BP-3), and OC make up a photostable UV filter combination (OC). The hydration and antiaging properties of these mixtures were studied using noninvasive biophysical techniques. Hairless mice were utilized in this experiment. As a consequence, both UV filter combinations did not influence the formulation’s hydration and anti-aging properties, including the vitamins tested in the surface and deeper layers, and reduced skin problems when the vitamins were contained in the formulation. Both UV filter combinations, i.e., photostable and photounstable, were found to boost vitamin A photostability research. Finally, the presence of UV filters may be considered advantageous for skin reduction [86].

#### 7.6.5. Bioactive Ingredient Rutin for Sunscreen (Antioxidant)

The photoprotective and antioxidant potential of rutin, which could enhance the SPF value while simultaneously giving sunscreen multifunctional capabilities, is currently generating much interest. Leticia Costa Tomazelli et al. improved the SPF of sunscreens by using the bioactive ingredient rutin. Skin disease can be caused by skin exposed to solar rays, and sunscreens are an important element in avoiding those undesirable outcomes. According to recent in vitro studies, rutin in sunscreens can exert antioxidant action while also increasing SPF. When rutin was present, a DPPH free radical scavenging assay showed a 40% improvement in radical scavenging capacity. Comparing the clinical SPF of rutin with UV filters to the formulation without bioactives, the clinical SPF was significantly increased to almost 70%. Because this is the first in vivo SPF assessment of a rutin-containing photoprotective preparation to be described in the literature, it is clear that rutin is a valuable and safe bioactive ingredient for use in multipurpose sunscreens [87].

### 7.7. Nanoemulsion of Soybean Oil, Avobenzone, and Octyl Methoxycinnamate

Vitamin E is present in soybean oil, which has antioxidant properties and naturally protects against the sun. In this context, Anayanti Arianto et al. fabricated a nanoemulsion of soybean oil, AVOB, and OMC to evaluate their synergistic effect on SPF. A high-energy emulsification method was used to prepare nanoemulsions containing AVOB (3%) and OMC (7.5%) with varying ratios of liquid paraffin and soybean oil. The prepared nanoemulsion was evaluated for different parameters, such as particle size, phase separation, SPF factor, physical stability, pH, and viscosity. The formulation, which contains 3% AVOB, 7.5% OMC with a ratio of 2.73% soybean oil, and 0.27% paraffin oil, has a droplet size of 68.47 nanometers, which is the smallest droplet size on average. There were 384.07 nm droplets in the formulation without soybean oil. The SPF value for the nanoemulsion containing soybean oil was 21.57 ± 1.21, while the value for the nanoemulsion containing no soybean oil was 16.52 ± 0.98. The AVOB nanoemulsion has been shown to be effective at synergistic sun protection with SPF. Compared to sunscreen emulsions, this sunscreen nanoemulsion was more stable at room temperature [88].

### 7.8. Surfactant and UV Filter

**Figure 7:**
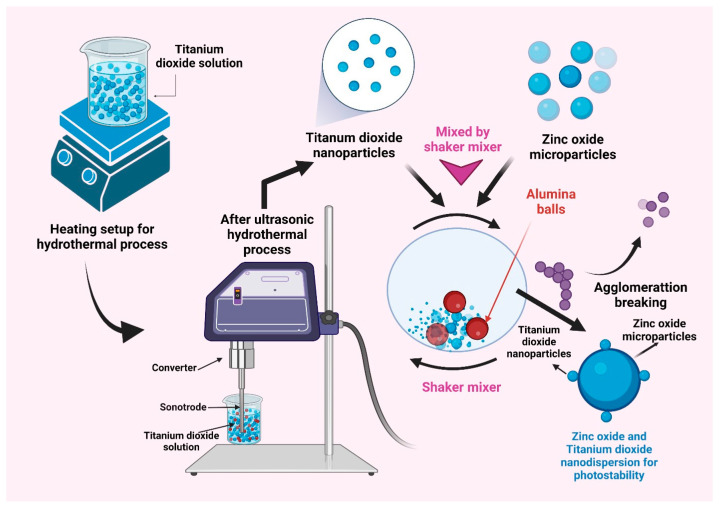
The nanodispersion of titanium dioxide nanoparticles and zinc oxide microparticles is achieved with a shaker mixer by using an alumina ball. Prior to mixing, titanium dioxide nanoparticles were prepared by using hydrothermal and ultrasonic treatment. The shaker mixer ensures agglomeration breaking and provides photostable nanodispersions ready for drug use.

The incorporated UV filters must be stable in the dispersion medium to achieve better stability. In this context, using the nanodispersion technique and experimental design, Haldi Ghasemiet et al. developed and optimized a sunscreen formulation containing surfactant and UV filters based on nanocomposite molecules. The continuous process was carried out in two investigations in this study due to the variety of elements impacting the end output (optimization of base cream and sunscreen). Six variables were explored in the first study, including surfactant nature (3 mixture variables), surfactant percentages, solvent, temperature, stability, and viscosity, which were assessed as responses. Using the exchange approach and *D*-optimality criteria, a multiplied model was assumed, and a set of 45 experiments was crossed. In another study, the Box-Behnken design was used to develop and analyze a set of 51 sunscreen tests based on photoprotective efficacy and photostability. These sunscreens include benzophenone 4, octyl salicylate, octyl methoxycinnamate, and AVOB, as well as a new nanocomposite that uses dry nanodispersion technology to disperse TiO_2_ nanoparticles over micrometric ZnO particles at variable ratios. The titanium dioxide particles were first passed through a hydrothermal process and later treated by an ultrasonic hydrothermal process to produce titanium dioxide nanoparticles. The prepared nanoparticles were mixed with zinc oxide microparticles by using a shaker mixer with the help of an alumina ball, which resulted in nanodispersion of the formulation containing titanium nanoparticles adsorbed on the surface of the zinc oxide microparticles, as shown in Figure 7.

According to the data, OMC has the greatest UVB protective effect when combined with other ingredients (SPF). UVA protection is improved by increasing the ratio of AVOB to ZnO. Organic UVA filters (AVOB) have a low photostability compared to organic UVB filters, which is relatively good. Inorganic filters are excellent photostable agents that improve the product’s stability. The novel dry nanodispersion ZnO-TiO_2_ composites provide increased UV coverage while reducing the overall number of nanoparticles required in sunscreens [89].

### 7.9. Prodrug System Use for Avobenzone and Diclofenac

Drug-induced photosensitization is a growing problem in society. Such an occurrence occurs when a chemical on the skin is exposed to sunlight. Since severe skin dermatitis has been associated with photosensitizing medications, sun protection is often advised. When applied externally, the nonsteroidal anti-inflammatory drug (NSAID) diclofenac is photosensitive. To make this topical treatment safer to use, researchers built and tested a unique pro-drug system that included diclofenac and AVOB. Through laser flash photolysis and phosphorescence emission studies, the dyad’s photoactive triplet excited-state features brought on by photosensitizing drugs were investigated. Finally, it has been demonstrated that AVOB protects diclofenac from photocyclization to carbazole compounds to some extent [90].

### 7.10. Nanocomplex (NCx) of ZnO Quantum Dots (QDs)

The sunscreen sector continues to face considerable challenges in finding appropriate multipurpose ultraviolet radiation (UVR) absorbents with great photostability, high molar absorptivity, widespread UVR screening, and desirable skin sensory properties. In this context, Adarsh Asok et al. created a nanocomplex (NCx) of ZnO quantum dots (QDs) by microwave synthesis in the presence of AVOB. It has been reported that this nanocomplex (NCx) showed the desired sunscreen properties. ZnO-QDs are the driving force behind the generation of highly photostable surface enolate species via the aldol condensation pathway, according to DFT and time-dependent DFT simulations of ZnO-AVOB hybrid structures and a comparison of their spectroscopic features with data. The measurements and experimental studies used in this paper can advance the development of photoprotection science and technology. Studies and computer models demonstrate that when AVOB interacts with ZnO-QDs, a highly photostable enolate form of AVOB develops on the exterior of the ZnO-QD. According to detailed spectroscopic studies, the production of NCx reduces the photochemical enol to keto transition in AVOB. Based on the reported theoretical and experimental results, this NCx can be employed as a potential multipurpose active ingredient for developing sunscreens with better barrier function [91].

### 7.11. Combination of Different UV Filters

The development of photostable sunscreens is critical for preserving UV protection and preventing photounstable filter reactive intermediates from acting as photooxidants when they come into direct contact with the skin. The goal of this research was to use HPLC analysis and spectrophotometry to assess the photostability of four distinct UV filter combinations in a formulation. The author examined four distinct UV filter combinations that are routinely found in SPF 15 sunscreen creams. OMC, benzophenone-3 (BP-3) and octyl salicylate (OS; formulation 1); OMC, AVOB and 4-methyl benzylidene camphor (MBC; formulation 2); OMC, BP-3, and OC (formulation 3); OMC, AVOB, and OC (formulation 4); OMC, AVOB, and OC (formulation 5); 40 mg of each formulation was spread on a glass plate, dried and then exposed to various UVA/UVB irradiation levels in photostability testing. The dried film was dissolved using ultrasound after immersion in isopropanol. HPLC analysis with detection at 325 nm and spectrophotometry were used to determine the filter components in the final solution. The 4 UV filter combinations studied in this study have varying photostability profiles, with formulation 3 (OMC, BP-3, and OC) being the best, followed by formulations 4, 1, and 2. OMC, AVOB, and BP-3 photostability were also improved by OC. The HPLC method presented was appropriate for determining the six UV filters in sunscreen at the same time. The extraction method worked well and was very precise. However, when the formulation was placed across the glass plate, a small quantity of it was lost, decreasing the accuracy of the extraction test [92].

### 7.12. Precipitation Method to Make Eu (Avobenzone) 3TPPO

Z. Wei et al. used the precipitation method to make Eu(AVOB)3TPPO. The thermal stability of Eu(AVOB)3TPPO was investigated using thermography and differential scanning calorimetry (TG-DSC). With an optimum excitation wavelength of 397 nm, a distinct emission wavelength of 612 nm, and color coordinates, Eu(AVOB)3TPPO exhibited a strong red characteristic fluorescence (0.66, 0.34). This formulation decomposes at approximately 200 °C, suggesting high thermal stability. This combination was successfully prepared and had excellent stability [93].

### 7.13. Organic UV Filters and Nanocarriers

When skin is exposed to sunlight, there is an alteration in the DNA of skin cells, and approximately 80% of cutaneous aging occurs. UV rays of sunlight directly affect skin pigments, so photoaging occurs. In this context, Luc Lo Pez-Hortas and colleagues investigated broad-spectrum photoprotective organic UV filters loaded with nanocarriers. To prevent photoaging, there is a need to develop sun protection products for personal care. The ability of sunscreen to block UVB and UVA radiation determines its photoprotective potential. As a result, sunscreen product formulations incorporate multiple wavelengths of UV filters to provide broad-spectrum skin protection. During the design and development of cosmetic formulations with photoprotective activity, many factors are considered, such as durability, water resistance, and type of vehicle. For UV protection, the photoprotective composition maintains a barrier layer on the skin. To avoid cell disruption, it should be photosensitive and not enter the epidermal layer. Moisturizing creams, lipsticks, makeup, and hair care products also incorporate UV filters to protect the hair structure from sunlight.

Based on their physicochemical properties, UV filters are classified into two groups.

- Inorganic or physical sunscreens:—These compounds reflect or scatter UV radiation and are available in the form of dry powders or dispersions. These compounds reflect UV A rays. It shows prolonged and stable photoprotection of the skin. Inorganic UV filters such as titanium dioxide are considered a possible carcinogen by the International Agency for Research on Carriers, especially when inhaled in large doses, which may enhance the propensity of cancer, as demonstrated in a rat model. Zinc oxide and titanium oxide have exhibited photocatalytic activity under certain conditions. After continuous exposure to UV radiation, inorganic UV filters such as titanium dioxide generate free radicals and thus help to accelerate issues of photomutagenesis, as tested in some in vitro studies.

Examples- ZnO, calcium carbonate, titanium dioxide.

**Figure 8:**
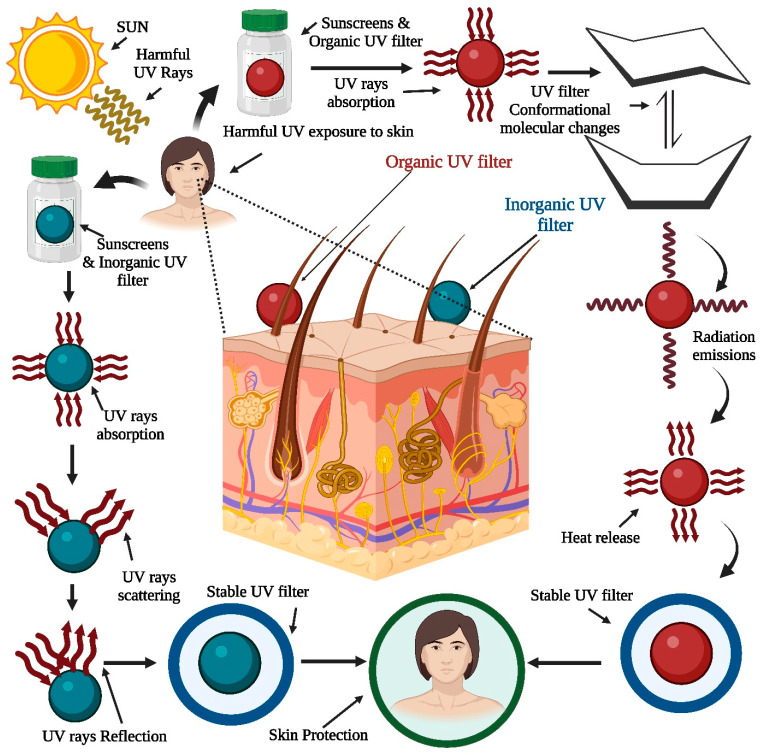
The interaction of organic and inorganic UV filters from sunscreen formulation with UV light. The organic UV filter undergoes conformational changes after UV exposure, followed by heat release. The organic UV filter works through the reflection, scattering or absorption of UV light.

Organic or chemical sunscreen: These filters absorb UV radiation and protect the skin. Their molar absorptivity in the UV region. These filters encourage the formation of melanin. Chemical sunscreens offer more extensive UVA and UVB protection than physical UV filters. These UV filters undergo a conformational change after UV exposure, followed by heat release. (Figure 8) UV filters are classified into three types based on the range of protection they provide: UVA, UVB, and UVC. This chemical also protects plastic food packaging materials from deterioration [94].

### 7.14. Challenges and Future Perspectives

For topically applied skin products, the stability of pharmaceutical actives is more important during the product life cycle, as the skin has a larger exposure to sunlight. This leads to photodegradation, and it can be intensified from longer-duration light exposure, leading to several complications induced by photodegraded products [29]. To protect such products, several strategies are used, including encapsulation, antioxidants, photostabilizers and the application of quenchers, as shown in Table 2.

The encapsulation of pharmaceutical actives is found to be a more promising photostabilization strategy, but it must be fueled by antioxidants and photostabilizers to develop a more impactful photostabilization strategy. There is a need to explore the clear mechanism for photodegradation in photosensitive drugs that leads to phototoxicity [99]. This can be useful for the selection of proper strategies in clubs for possible synergistic effects and to counter multiple pillars of photoprotection. This includes the enhancement of photostabilization by encapsulation and photostabilization with minimal detrimental effects induced by reactive oxygen species by antioxidants. To ensure proper photostabilization, several other encapsulating polymers apart from cyclodextrin can be used, such as ethyl cellulose and Eudragit combined with antioxidants originating from polyphenols such as lutein. There is a need to develop effective quenchers compatible with photosensitive drugs and other strategic materials to frame possible gold-standard therapies to address photoprotection issues. The entrapment efficiency of photostabilizers such as octocrylene loaded with UV filters such as avobenzone needs more exploration and upgradation in combination with antioxidants and quenchers. There is also a scope to investigate the impact of strategies on the delivery of UV filters by varying several processing factors, formulation factors and materials used for the same [29,99,100].

## 8. Skin Absorption of Avobenzone

The use of highly soluble UV filter carriers, chemical alterations to enhance their molecular weight, complex formation with cyclodextrins, or integration in microparticles are methods used to prevent or reduce sunscreen absorption through the skin. In this context, S. Scalia et al. investigated the effect of cyclodextrin on butyl methydibenzoylmethane absorption by human skin in vitro. For this study, the authors used hydroxypropyl-cyclodextrin (HP—CD) and sulfobutylether—CD (SBE7—CD). The reaction between the UV filter and the cyclodextrins in water was investigated using phase-solubility analysis. Extracted human skin was placed on Franz diffusion cells with AVOB free or complexed with cyclodextrin solutions, and the amount of sunscreen that penetrated the stratum corneum, viable epidermis, dermis, and receptor fluid after 6 h was evaluated using HPLC. The administered dose of BM-DBM penetrated 14.10–16.78% of the cutaneous tissue. There was no sunscreen in the dermis or recipient phase. The stratum corneum contained the majority of the absorbed UV filter (84.6–95.5%), with no differences between uncomplexed and complex AVOB. In the preparation containing free BM-DBM, the sunscreen ingredient was found in considerable amounts in the epidermis (2.29% of the applied dose). Complexation with SBE7—CD significantly lowered the epidermal concentration of the UV filter (0.66% of the applied dosage), whereas HP—CD had no impact. SBE7—CD reduces BM-DBM skin penetration [101].

### Human Skin Model for the Phototoxicity Study of Sunscreen Formulations

In vitro, three-dimensional human skin models are a novel method for assessing cytotoxicity and phototoxicity in the cosmetics sector. Dayane P. Luco et al. used a three-dimensional human skin model for the phototoxicity study of sunscreen formulations. The author’s study aimed to determine the possible toxicity of formulations containing UV filters with and without UV exposure using this model. The toxicity of these formulations was also assessed when they were exposed to photodegradation. The results showed that AVOB was more unstable and hazardous than octyl p-methoxycinnamate under identical test conditions. Some unirradiated formulations showed hazardous effects. This research highlights the need to put sunscreen formulations to the test in real-life situations. Finally, this noninvasive technique might be used to screen new sunscreen products rather than employing animal models in general for phototoxicity testing [102].

## 9. Persistent Pigment Darkening Method (PPD)

The effectiveness of sunscreen UVA protection can be evaluated using the persistent pigment darkening (PPD) method. PPD in vivo has been suggested as a measure of UVA protection. The researchers wanted to see if PPD could reliably assess the in vivo efficiency of UVA filters both alone and in combination with UVB filters. Sun protection efficacy has been tested in many marketed photoprotective lotions. UVA protection increased in the lockstep with UVA filter concentration and was unaffected by UVB filters [103]. The PPD technique was found to be responsive to all UVA filters. The researchers observed that evaluating UVA protection in vivo by the persistent pigmentation component (PPD, quantified 2–4 h postexposure) is a valid predictor of UVA protection. According to the authors, the PPD UVA-PF test method is sensitive to UVA filter concentrations. It is unaffected by the presence of UVB filters. According to research, commercial sunscreens with the same UVB protection efficiency (SPF 15 or 30) have different UVA protection levels. As a result, independent UVB and UVA measurements must be used to assess the efficacy of sun protection solutions [104].

## 10. Toxic Effects of UV Filters

The estimation of the toxic effect of various UV filters on the environment is essential to understand the safe concentration of UV filters for the formulation of sunscreen products. In this context, Xin Zhong et al. experimented on cucumber plants to compare the toxicity of different filters. Chemical sunscreens frequently contain UV filters such as oxybenzone (OBZ), AVOB (AVOB), OC, and octinoxate (OMC) [105]. UV filters damage the environment from different human-made sources, such as sewage discharge. The research discovered that irrigating cucumber plants with water that presented these four UV filters notably inhibited their aboveground development in this investigation. Photosynthesis was suppressed by nonstomatal components in all UV filters, although in varied ways [106]. Only OBZ directly inhibited photosynthesis; the others (AVB, OCR, and OMC) inhibited photosynthesis through the Calvin-Benson cycle [107]. Long-term use of these four UV filters reduced plant respiration because of the formation of reactive oxygen species, which directly affected the production yield. In addition, the study discovered that these four UV filters caused cucumber plants to suffer from varying degrees of phototoxicity [108]. Based on a detailed investigation, the toxicity of these UV filters was in the following order: OBZ > AVOB > OMC > OCR. The findings of this study may serve to enhance consumer and industry awareness of the hazards of UV filters in cosmetics and over-the-counter medications, as well as persuade consumers and industry to reduce or remove their use [109,110].

Most chemical formulations used for sunscreen agents are lipophilic and alcohol, which contributes to better drug delivery to the skin. Several approaches have been used to combat the photodegradation of photosensitive drugs by using encapsulation, quenchers, antioxidants, etc. Antioxidant agents designed with nanoparticle drug delivery systems have made a major contribution to apoptosis [111].

Chang Beom Park et al. performed a study to test the toxicity of UV filters on Daphnia magna. Aquatic creatures are commonly exposed to combinations of numerous chemical compounds in freshwater habitats. The toxicity of three organic UV filters (ethylhexyl methoxycinnamate, OC, and avobenzone) to Daphnia magna was examined in this study to assess their cumulative toxicity when used together. Based on these findings, researchers hypothesized that UV filters interact with one another to alter the toxicity mechanisms inside D. manga. The combined effects of binary or ternary mixes might be validated as antagonistic using CA- and MDR-based techniques. These component interactions may be influenced by the concentration combination or relative potency of the components in a mixture. The outcomes of this study, according to the authors, will be valuable in the application of chemical mixture management for environmental health protection [112].

## 11. Clinical Study of Sunscreen Active Ingredients

Sunscreen active agents with issues of systemic absorption through skin greater than 0.5 ng/mL are considered for testing by the United States Food and Drug Administration (USFDA) department. The US FDA has given guidance that such formulations with systemic absorption safety concerns need to have nonclinical toxicological testing. These tests should also include systemic carcinogenicity along with reproductive studies. Sunscreen products are considered over-the-counter (OTC) products in the USA, and such agents are used as major cosmetic products. Since the majority of the systemic absorption scenarios of OTC products are not known, there is a need to explore this area for the amount of sunscreen agents to be systemically absorbed.

The USFDA has recommended the assessment of the systemic absorption of sunscreen through sunscreen guidance named “Guidance for Industry: Nonprescription Sunscreen Drug Products Safety and Effectiveness Data.” This study also explored a nonclinical safety assessment covering two different studies. These studies are about dermal carcinogenicity along with embryofoetal toxicity. Three clinical trials have been completed for avobenzone. The details are presented in Table 3 [113].

Murli K. Matta et al. studied the plasma levels of UV filters in a randomized clinical trial. Sunscreen was administered to 75% of the body surface area at a rate of 2 mg/cm^2^. They used 6 active ingredients. All 6 active ingredients exceeded a plasma concentration of 0.5 ng/mL on day 1 after a single application. A rash developed in 14 participants as the most common adverse event [114,115].

Kenji Atarashi et al. used butyl methoxy dibenzoylmethane to reduce ketoprofen photoallergic reactions by adding it to the topical ketoprofen formulation. Allergic contact dermatitis is induced by the topical administration of ketoprofen (KP). To avoid this side effect, the author looked at the benefits of each ultraviolet (UV) filter used in topical ketoprofen formulations [116].

## 12. Conclusions

The novel ultraviolet A blocker AVOB has shown a significant role in blocking ultraviolet light. Ultraviolet A light has many harmful effects on the skin. Different approaches are used to solve the stability problem of UV filters. Cyclodextrin (CD) is the most commonly utilized encapsulation technology, followed by liposome lipid nanoparticles, microparticles and polymeric nanoparticles. Choosing a photo stabilization strategy involves researching the photodegradation mechanism, assessing the final product’s effectiveness of photo stabilization, and comparing alternative strategies to look for the best one from a cost-benefit perspective. Finally, assurance of the pharmaceutical product’s efficacy should be highlighted. In the future, photostability experiments performed with a solar radiation exposure environment may become more valuable, allowing for easier comparisons. There is still scope to try out different encapsulating polymers, such as ethyl cellulose and eudragit, for photoprotection along with the combination of other strategies, such as the use of antioxidants and photostabilizers, as a synergistic effect for more effective photostabilization with enhanced SPF. Understanding photodegradation pathways is a crucial step in the design of pharmaceuticals. The combination of several strategies, including encapsulation, antioxidants, photostabilizers and quenchers, can be used as an effective tool for photoprotection.

## Figures and Tables

**Table 1:** Avobenzone Properties.

IUPAC Name	1-4-(*Tert*-butyl phenyl)-3-(4-methoxyphenyl) propane-1,3-Dione
Class	Dibenzoyl methane molecules
Other Names	Butyl methoxydibenzoylmethane (BMDM), Avobenzone
Trade Name	Escalol 517, Eusolex 9020, Parsol 1789 and NeoHeliopan 357.
Empirical Formula	C_20_H_22_O_3_
Molecular Weight	310.39 g/mol
Color And Odor	Yellow powder and weak characteristic odor.
Solubility	soluble in organic solvents like DCM, Methanol Water-insoluble (0.01 mg/L at 20 °C).

**Table 2:** Summary of the photostabilization strategy mechanisms.

Photostability Strategies	
Description	Photostabilization Mechanism
**Solar Filters**	
UV filter (organic, inorganic, and broadband)	By overlapped spectra, photosensitive chemicals are protected. Inorganic filters can reflect or scatter UV and VIS radiations, providing an impenetrable barrier, while organic filters possess functional groups that can absorb UV radiation [4].
**Antioxidants**	
Molecules with the capacity to take up or give electrons to the unpaired free radicals, neutralizing them as a result and inhibiting the oxidation of the substrate.	Excited state quenching through energy transfer, charge transport, or interaction with free radicals (scavengers) [95]. Antioxidants may exert a biologically meaningful filter capacity since they are involved in the chemical stability of photosensitive substances and, in some situations, by absorbing UV-R [96].
**Encapsulation**	
Hydroxypropyl-*β*-cyclodextrin (HPCD)	Their molecular makeup enables them to enclose a variety of substances into their hydrophobic reservoir, creating a barrier against light and other environmental influences [59]. There is less skin penetration and photodecomposition of avobenzone with enhanced photoprotection activity through cyclodextrin encapsulation [72].
Encapsulation into lipidic carriers	Changes in the makeup of these lipid-based systems enable changes in their transmission properties would influence the scattering of the radiation and the amount of light scattering. Approximately 70% of molecular sunscreen was loaded into lipidic carriers to achieve high SPFs [97].
Encapsulation into polymeric carriers	UV filters encased in polymethylmethacrylate have improved photoprotection, photostability, and safety [63]. Sunscreen lotions containing morin-loaded polymeric NPs were found to have excellent skin penetration in in vitro and in vivo skin penetration studies [62].
Encapsulation into inorganic nanocarriers	Silica is an excellent candidate for a carrier material due to the presence of mesoporous pores [98]. Due to the large surface area and porous volume, 90% of the sunscreen agent was encapsulated effectively with excellent UV protection [75].
**Coencapsulation of Sunscreen Agent with Antioxidant into A Suitable Carrier**	
Octocrylene sunscreen and flavonoid (luteolin) into SLNs	The results obtained showed a sun protection factor higher than 30, resulting in a higher photoprotection capacity [99].
Zinc oxide, octocrylene, and quercetin encapsulated nanoparticles	The in vitro SPF for formulations containing ZnO NPs was 29 ± 5 with 80–92% of encapsulation efficiency [100].

**Table 3:** Clinical trial details for avobenzone.

Title	Sponsor	Collaborator	Study Type	Study Phase	Study Design	ClinicalTrials.Gov Identifier	Recruitment Status
Determination of Sun Protection in Sunscreen Formulas (Study SR09-15; P08236; COMPLETED; PFA and SPF)	Bayer	-	Interventional	Phase 3	Non-Randomized	NCT01001975	Completed & last updated on 11 March 2015
Assessment of the Human Systemic Absorption of Sunscreen Ingredients	Food and Drug Administration (FDA) USA	Spaulding Clinical Research LLC USA	Interventional	Phase 1	Randomized	NCT03582215	Completed & last updated on 21 April 2020
Ultraviolet and UV—Visible Light Photoprotection for the Treatment of Melasma	Universidad Autonoma de San Luis Potosí	Hospital Central “Dr. Ignacio Morones Prieto”	Interventional	Phase 4	Randomized	NCT01695356	Completed & last updated on 2 December 2014

## Data Availability

No new data were created or analyzed in this study. Data sharing is not applicable to this article.
